# Switchable aqueous catalytic systems for organic transformations

**DOI:** 10.1038/s42004-022-00734-z

**Published:** 2022-09-26

**Authors:** Nikita Das, Chandan Maity

**Affiliations:** Department of Chemistry, School of Advanced Sciences (SAS), Vellore Institute of Technology (VIT), Vellore, 632014 Tamil Nadu India

**Keywords:** Organocatalysis, Self-assembly

## Abstract

In living organisms, enzyme catalysis takes place in aqueous media with extraordinary spatiotemporal control and precision. The mechanistic knowledge of enzyme catalysis and related approaches of creating a suitable microenvironment for efficient chemical transformations have been an important source of inspiration for the design of biomimetic artificial catalysts. However, in “nature-like” environments, it has proven difficult for artificial catalysts to promote effective chemical transformations. Besides, control over reaction rate and selectivity are important for smart application purposes. These can be achieved via incorporation of stimuli-responsive features into the structure of smart catalytic systems. Here, we summarize such catalytic systems whose activity can be switched ‘on’ or ‘off’ by the application of stimuli in aqueous environments. We describe the switchable catalytic systems capable of performing organic transformations with classification in accordance to the stimulating agent. Switchable catalytic activity in aqueous environments provides new possibilities for the development of smart materials for biomedicine and chemical biology. Moreover, engineering of aqueous catalytic systems can be expected to grow in the coming years with a further broadening of its application to diverse fields.

## Abstract (web summary)

Control over chemical transformations in aqueous environments employing catalytic systems whose activity can be switched on/off is challenging. Here, the authors review the switchable catalytic systems that operate in aqueous environments in response to external stimuli, such as pH, temperature, light, small molecules, electric field, magnetic field and mechanical energy.

## Introduction

The enzymes in natural biosynthetic processes work in aqueous environment, where they reach amazing levels of efficiency, and selectivity. Enzyme catalysis is often used in nature to control formation of molecules and/or complex structures for achieving homeostasis, motility, and signaling processes. The catalytic processes generally take place in parallel, and the required spatiotemporal control of the catalytic activity often entails its modulation by physicochemical stimuli such as small molecules, light, temperature, and pH^1^. Stimuli-induced enzyme catalysis and the underlying mechanism have been an important source of inspiration for the design of biomimetic artificial catalysts. Scientists have devoted many efforts to obtain artificial catalytic systems in aqueous environment with the aim of mimicking the levels of performance of natural enzymes^2,3^. However, the artificial catalysis usually takes place according to the initially chosen reaction conditions, and lacks the control over the chemical process^4^. The rate of chemical transformations can be modulated employing stimuli-triggered catalysts having stimuli-responsive features in the structure of the catalytic systems^5–8^.

In aqueous media, the artificial switchable catalysis could be a key factor to develop more efficient nature-inspired catalytic systems for the green industrial processes^9,10^. At this regards, organic transformations have been studied in water as the reaction medium^11–14^. Water possesses many desirable characteristics as reaction medium due to its environmental friendliness, high polarity, large cohesive energy, high heat capacity, and hydrogen bonding abilities that may influence reaction rate and selectivity^12,15^. Besides, use of water as reaction medium benefits chemical processes by allowing mild reaction conditions, simplifying operations, and sometimes providing unforeseen reactivities and selectivities^13,16^. Despite this, organocatalytic reactions in aqueous environments encounter considerable challenges such as poor reactant solubility, transition states destabilization due to disturbance of hydrogen bonding interactions, and poor hydrolytic stability of catalytic intermediates or chemical species^17,18^. Over the past few decades, attempts have been made to overcome these challenges for various organic transformations in aqueous media^14,19–22^. However, achieving reversible control over chemical transformations in aqueous environment remains a challenge.

There have been few studies devoted to the controlled organic transformations in aqueous environment employing switchable catalytic systems, whose catalytic activity can be switched ‘*on*’ and ‘*off*’ by stimuli. These systems are promising candidates for the application in biological environments and/or industrial processes. Generally, these catalyst systems have smart structural features that respond reversibly according to the presence/absence of stimuli for speeding up or slowing down the reaction rate. The switching mechanism of these systems mostly depends on (i) accessibility of the active sites of catalytic system that can be modified by the stimulus due to change in electronic, steric, or cooperative effects in the systems, and (ii) availability of suitable microenvironment via aggregation/dissociation of the catalytic system in presence/absence of external stimulus. For example, temperature-, pH-responsive catalytic systems usually change the aggregation state for suitable microenvironment in presence/absence of stimuli, whereas light-, small molecule-responsive catalytic systems depend on the electronic, steric, or cooperative effect of the system for accessing the active site of the catalyst.

**Fig. 1:**
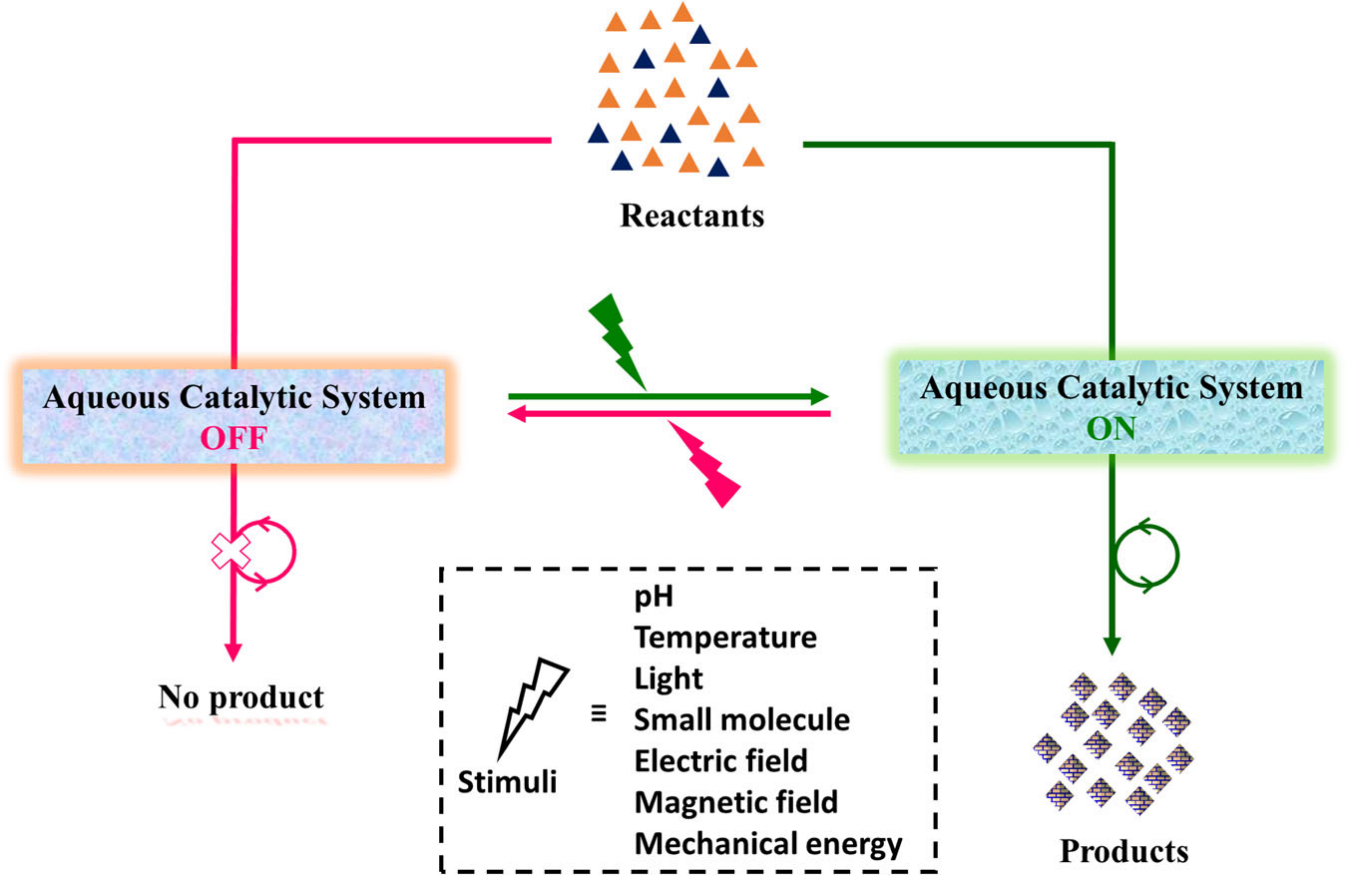
Switchable aqueous catalytic system. A schematic representation of switchable catalytic systems in aqueous environments. Presence or absence of trigger can module the catalytic activity in switch *‘on*’/‘*off*’ mode for organic reactions.

In this review, we describe stimuli-induced artificial switchable catalytic systems with classification in accordance to the stimulating agent (Fig. 1). The scope of this review encompasses organic reactions in aqueous buffer solution or water/organic solvents mixture. We discuss the state-of-the-art and identify the challenges in this field. We describe “in water”^14^ stimuli-switchable systems, where the reactants, catalyst, and products are present in aqueous (buffer) solution. We also describe “on water”^20^ stimuli-switchable systems, which comprise emulsions, hydrogels, and supramolecular assemblies or immobilized catalyst on solid support, where water exerts a critical effect on the reaction rate. However, biocatalysis^23–25^ for organic reactions in aqueous medium have not discussed here as they involve enzyme engineering, and are not fully synthetic systems. Altogether, switchable catalysis for organic transformations in “*nature like*” environment would find applications in smart materials such as self-healing materials, controlled delivery, spatiotemporal on-demand drug synthesis, therapeutics, and soft robotics. The examples of different chemical transformations have summarized in Table 1, and this article is divided according to the stimulating agent employed for switchable catalytic systems, which we now describe.

**Table 1:** The summary of switchable catalytic systems for chemical transformations in aqueous environments.

Entry no.	Chemical transformations	Catalyst moiety, Medium^Reference^	Stimuli	Catalysis status, Yield
1	Ester hydrolysis	Histidine, Tris-HCl buffer^39^	pH = 9.0	ON, -^N.R.^
			pH = 6.0	OFF, -^N.R.^
2		Imidazole, Water^73^	*T* > 32 °C	ON, -^N.R.^
			*T* < 32 °C	OFF, -^N.R.^
3		Cavity of β-CD, Tris buffer (pH 8.7)^113^	UV light	ON, -^N.R.^
			Visible light	OFF, -^N.R.^
4		Imidazole, PBS buffer (pH 7.2)^115^	UV light	ON, -^N.R^
			Visible light	OFF, -^N.R.^
5		histidine, PBS buffer (pH 7.4)^117^	Visible light	ON, -^N.R.^
			UV light	OFF, -^N.R.^
6		Imidazole-Zn complex, PBS buffer (pH = 7.6)^122^	UV light	ON, -^N.R.^
7			Visible light	OFF, -^N.R.^
8	Aldol reaction	Isoindoline, Water^40^	pH > 4.5	ON, 99%
			pH < 5	OFF, 0%
9		L-proline, Water^50^	pH = 4.0	ON, 81% (ee)
			pH = 7.0	OFF, 2% (ee)
10		L-proline, Water^51^	pH = 7.0	ON, 89% (ee)
			pH = 4.0	OFF, 37% (ee)
11		L-proline, Water^72^	*T* = 50 °C	ON, 95%
			*T* = 25 °C	OFF, 53%
12		L-prolineamide, Water^74^	*T* = 40 °C	ON, 83%
			*T* = 25 °C	OFF, 10%
13		L-proline, Water^80^	*T* = 30 °C	ON, 96% (ee)
			*T* = 80 °C	OFF, 12% (ee)
14		L-proline, Water^134^	CO_2_ (5 Mpa)	ON, 99%
			No CO_2_	OFF, 0%
15		L-proline, Water^176^	*T* = 50 °C	ON, 94%
			*T* = 25 °C	OFF, 79%
16	Michael reaction	Isoindoline, Water/THF^40^	pH = 6.7	ON, 23%
			pH = 3.6	OFF, 0%
17	Suzuki reaction	Pd-NP, Water^56^	pH > 7.0	ON, >94%
			pH < 7.0	OFF, No reaction
18	Reduction of nitrophenol	Au-NP, Water^57^	pH = 5.0	ON, -^N.R^
			pH = 9.2	OFF, -^N.R^
19		PBT, Water^173^	CO_2_ & light,	ON, 96%
			N_2_	OFF, 0%
20	Hydrogenation of styrene	Pd-NP, Water/toluene^62^	pH = 3.0–4.0	ON, 99%
			pH = 7.0–8.0	OFF, 0%
21		Pd, Water^128^	Visible light	ON, >99%
			UV light	OFF, 0%
22	Acrolein oxidation	Active Se, Water/dioxane^76^	*T* = 50 °C	ON, >90%
			*T* = 20 °C	OFF, No reaction.
23	Acetylation	DMAP, Water^81^	*T* = 5 °C	ON, 98%
			*T* = 50 °C	OFF, No reaction
24	Click reaction	Cu(PPh_3_)_2_NO_3_, Water/organic reagent bilayer^82^	*T* < 32 °C	ON, -^N.R^
			*T* > 32 °C	OFF, No reaction
25	Decomposition of hydroperoxide	Ph_3_CPF_6_, Water/organic reagent bilayer^82^	*T* < 32 °C	ON, -^N.R^
			*T* > 32 °C	OFF, No reaction
26	Hydrosilylation	H_2_PtCl_6_, Water/organic reagent^82^	*T* < 32 °C	ON, -^N.R^
			*T* > 32 °C	OFF, No reaction
27	Dechlorination	Pd-NP, Water/toluene^97^	*T* < 32 °C,	ON, 99%
			*T* > 32 °C,	OFF, No reaction
28	Hydrogenation of alkene	Ru-NP, Water^105^	*T* > 39 °C	ON, -^N.R^
			*T* < 39 °C	OFF, No reaction
29	Racemization	Aldehyde and pyridinium group, D_2_O/CD_3_CO_2_D^112^	UV light (*λ* = 365 nm)	ON, 95%
			Visible light (*λ* > 490 nm)	OFF, 3%
30	Hydrolysis of glycopyranoside	Biscarboxylic acid, Water^119^	UV light (*λ* = 365 nm)	ON, -^N.R.^
			Visible light	OFF, -^N.R.^
31	Transphosphorylation	Zn(II)-based catalyst, HEPES buffer (pH 7.0)^123^	Visible light	ON, -^N.R.^
			UV light (*λ* = 365 nm)	OFF, -^N.R.^
32		Zn(II)-based catalyst, HEPES buffer (pH 7.0)^124^	UV light (*λ* = 365 nm)	ON, -^N.R.^
			Visible light (*λ* = 465 nm)	OFF, -^N.R.^
33		Zn(II)-based catalyst, Pseudo-aqueous solution^140^	Presence of CO & Cl^−^	ON, 100%
			Absence of CO and Cl^−^	OFF, No reaction
34		Cu(II)-based catalyst, HEPES buffer (pH 7.0)^170^	Oxidative potential	ON, -^N.R.^
			Reductive potential	OFF, -^N.R^
35	Hydrolysis of acetal	Acid catalysis in ‘nanoflask’, Water-saturated toluene^127^	UV light (*λ* = 365 nm)	ON, ~85%
			Visible light	OFF, ~55%
36	Oxidation	Cu(I)-bpy & TEMPO, Aqueous borate buffer (pH 9.5)/acetonitrile (1/1)^143^	ssDNA antitrigger sequence	ON, -^N.R^
			ssDNA trigger sequence	OFF, No reaction
37		Aneli system, Water/organic substance^177^	No CO_2_	ON, >94%
			CO_2_ & magnetic field	OFF, No reaction
38	Hydrazone formation	Aniline, PBS buffer (pH 7.5)^165^	Glycine betaine methyl ester	ON, -^N.R^
			Ester hydrolysis	OFF, -^N.R^
39	Oxidation of furoic acid	PBT, Water^173^	CO_2_ & light	ON, >99%
			N_2_	OFF, No reaction
40	Arylation	PBT, Water^173^	CO_2_ & light,	ON, 85%
			N_2_	OFF, No reaction

*T* temperature, *-*^*N.R*^ not reported, *CD* cyclodextrin, *PBS* phosphate-buffered saline, *HEPES* 4-(2-hydroxyethyl)-1-piperazineethanesulfonic acid, *Tris* tris(hydroxymethyl)aminomethane, *IEP* isoelectric point, *ILS* ionic liquid surfactant, *TACN* 1,4,7-triaza-cyclononane, *TEMPO* 2,2,6,6-tetramethylpiperidine-1-oxyl, *NP* nanoparticle, *PBT* Poly(bisthiophene).

## pH-induced switchable catalytic systems

Change in pH in aqueous solution can substantially change the property of the medium and physicochemical responses of the species that present in the solution. In nature, many enzymatic reactions are pH-sensitive^26–29^. pH stimulus can regulate the catalytic activity of natural enzymes, where the conversion of substrate to product takes place within the microenvironment of a catalytic site. As an example, triosephosphate isomerase (TIM) is a crucial enzyme in the glycolytic pathway, which catalyzes the reversible isomerization of D-glyceraldehyde 3-phosphate to dihydroxyacetone phosphate. The forward and reverse reactions catalyzed by TIM is pH dependent^30^. At the active site of TIM, proton transfer from dihydroxyacetone phosphate to glyceraldehyde phosphate is carried out by the carboxylate side-chain of Glu165/167^31^ and by the imidazole side-chain of His95^32^.

**Fig. 2:**
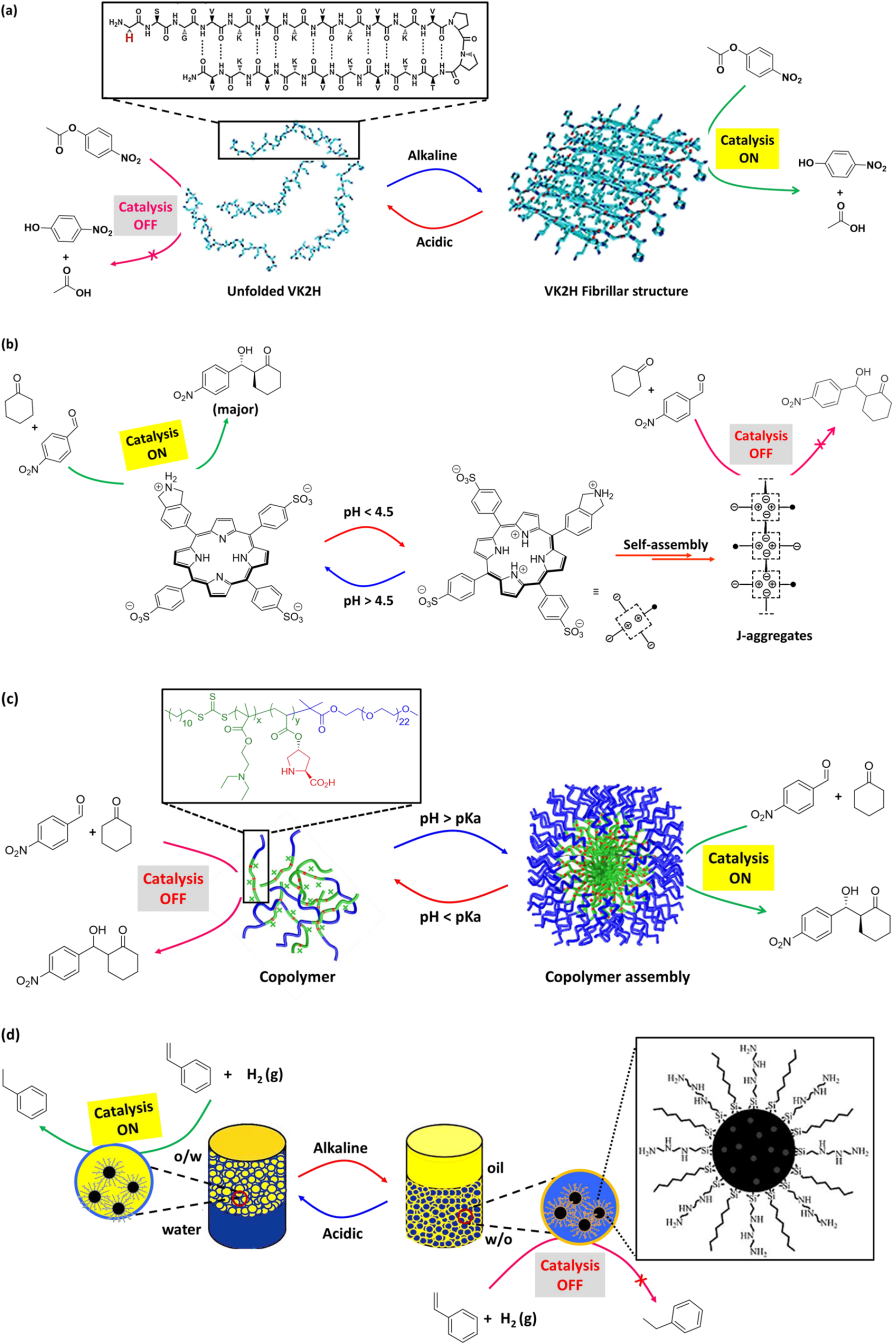
pH-switchable catalytic systems. **a** Schematic representation of the pH-switchable VK2H peptide as artificial hydrolase. The peptide showed conformational transition from unfolded random coil to β-sheet via changing the pH from acidic to alkaline. (Inset: chemical structure of peptide VK2H). The fibril structure can catalyze the hydrolysis of pNPA, whereas the unfolded structure is catalytically inactive. Switching the catalytic activity can be controlled by altering the pH. Adopted with permission from ref. ^39^, copyright 2017 Wiley-VCH Verlag GmbH & Co. KGaA, Weinheim. **b** pH-switchable organocatalysis with amine-porphyrin hybrid in aqueous solution. Formation of J-aggregates, at acidic pH, suppressed the catalytic activity of Isoindoline moiety of the hybrid, whereas deaggregated state of the hybrid at higher pH resulted efficient aldol reaction. Adopted with permission from ref. ^40^, copyright 2020 WILEY-VCH Verlag GmbH & Co. KGaA, Weinheim. **c** A schematic of assembly behavior of polymer structure in aqueous solution at different pH (inset: the structure of the polymer), and aggregation-induced aldol reaction that catalyzed by proline moiety. Adopted with permission from ref. ^51^, copyright 2020 Elsevier B.V. **d** Schematic of pH-induced emulsion inversion for styrene hydrogenation. At acidic pH, the catalyst can efficiently convert the substrate to product, whereas the reaction is terminated at basic pH (inset: The structural description of silica microsphere with catalytically active center). Adopted with permission from ref. ^62^, copyright 2013 WILEY-VCH Verlag GmbH & Co. KGaA, Weinheim.

In comparison to natural enzymes, it is difficult to replicate the complex enzymatic environment for artificial systems. Incorporation of pH-responsive features in artificial supramolecular systems, pH-triggered catalytic activity can be realized^33–38^. However, pH-induced switching of catalytic activity due to aggregation/dissociation of supramolecular systems remains relatively unexplored. In an example, Zhang et al.^39^ reported pH-switchable artificial hydrolase activity, where a catalytic histidine residue was introduced at the edge of a pH-responsive peptide, VK2H (Fig. 2a). By changing pH from acidic to alkaline, the peptide exhibited a conformational transition from random coil to β-sheet. The β-sheet self-assembled to long fibrils, where histidine residues extended in an ordered array as an effective catalytic microenvironment. The fibrils showed significant esterase activity by catalyzing the hydrolysis of *p*-nitrophenyl acetate (pNPA) (kinetic efficiency = 19.18 s^−1^ M^−1^) in tris-HCl buffer (Table 1, Entry 1). The role of histidine as catalytic site was confirmed by replacing histidine residue of the peptide with glycine (VK2G), which self-assembled into fibrils, but with much reduced catalytic activity. Besides, the peptide formed a hydrogel at higher concentrations, which is also catalytically active. In the self-assembled form, the histidine residues were exposed on the edges of the fibril, which provided the active site for substrate binding. Switchable catalytic activity was realized by pH-induced disassembly of the fibrils into random coils. Changing the pH between acidic (pH 6.0) and basic (pH 9.0), the phase behavior can be reversibly realized from gel to fluid, where the unassembled random coils were catalytically inactive.

Employing amphiphilic molecule, pH-Induced modulation of catalytic activity of a supramolecular system was demonstrated by Arlegui et al.^40^ (Fig. 2b). In this example, supramolecular system showed catalytically inactivity in aggregated state of the amphiphile, whereas catalytic activity was realized at dissociated state. The catalytic activity of cyclic secondary amine was demonstrated in an amphiphilic meso-(4-sulfonatophenyl)porphyrin derivative by adjusting the homogeneity of the aqueous solutions via pH change. In neutral aqueous solutions, the highly soluble free-base form of the porphyrin derivative showed enamine-based organocatalysis. In contrast, the porphyrin core was protonated under acidic conditions, and led to the formation of self-assembled structures. The protonation of the central pyrroleninic core at acidic pH (typically below pH 4.8) induced the formation of J-aggregates, which was stabilized by ion-pair contacts between the cationic porphyrin centers and the peripheral anionic sulfonate groups. As a result, porphyrin-based pyrrolidine derivatives flocculate and the catalytic activity is fully suppressed. There is no aldol product formation even after 8 days at acidic pH (pH = 3.6) (Table 1, Entry 8). In comparison, the catalysis of the aldol reaction between cyclohexanone and p-nitrobenzaldehyde (pNB) took place by isoindoline moiety with excellent yield (99%) and selectivity (93:7 anti:syn mixture of the aldol products) in the solution of higher pH value (pH = 6.7). However, at acidic pH, the catalyst aggregates can be easily separated from the reaction products via centrifugation. Thereafter, neutralization and desalting could provide the sulfonated amine-porphyrin hybrids, which retains their catalytic activity, and can be reused. Though, there is a substantial decrease of catalytic activity after few cycles.

Besides aldol reaction, isoindoline-functionalized amphiphilic porphyrin derivative was employed for Michael reaction between cyclohexanone and 2-nitrostyrene. However, poor yield (23%) of Michael adduct was reported even after three days in the solution of higher pH value, whereas no conversion was reported at lower pH value (Table 1, Entry 16). Nevertheless, the aggregation state of amphiphilic porphyrin derivatives and their catalytic activity can be controlled by means of pH stimulus. It is worth noting that self-assembled state of supramolecular systems can switch catalysis *‘on*’ or ‘*off*’ depending on availability of suitable active-site microenvironment for the reactant(s) and favorable effect of the system to access the active-site for catalysis. For example, cooperative effect favors pNPA hydrolysis^39^ in self-assembled state of V2K2H, whereas self-assembled state of amphiphilic porphyrin derivative^40^ flocculates and therefore substrates of aldol reaction cannot access the catalytic sites, resulting switching ‘*off*’ the catalysis.

Polymeric materials, in response to pH stimuli, can undergo structural and/or property change, such as surface activity, chain conformation, solubility^41^. Immobilization of organocatalyst moiety within such polymer scaffolds may result in formation of aggregates with desired microenvironment, where efficient and selective organic reactions could be possible^42^. In this regard, proline is used as one of the popular organocatalyst that facilitate chemical transformations similar to type 1 aldolase enzyme catalysis^43,44^. Proline-catalyzed aldol product can be formed in the hydrophobic microenvironment through an enamine mechanism, where water content at the catalytic site has an impact on the selectivity of the final product. Higher reaction rates and improved (stereo)selectivity could be obtained in the presence of small amount of water, whereas high water concentration can significantly lower the conversions and selectivities^19,45^. Moreover, proline functionalized with nonpolar moieties demonstrated the importance of hydrophobic environment for proline-catalyzed aldol reaction^46,47^. Proline can be anchored to polymer scaffolds for asymmetric aldol reactions with a range of ketones and aldehydes in aqueous environment^48,49^. In one example, Prado et al.^50^ employed an amphoteric alternating copolymers of hydrophobic phenylmaleimide and hydrophilic vinylpyrrolidone for supporting L-proline-catalyzed aldol reaction in aqueous buffer solution (Table 1, Entry 9). It is reported that near the isoelectric point (IEP) of the polymer (around pH 4), an aggregated form of the polymer scaffold favored the formation of high enantioselective product (91% yield, 81% ee). This high enantioselectivity is attributed to the exclusion of water from the polymer aggregates, resulting stabilization of the transition state. In contrast, the hydrophilicity of the aggregates increased at higher pH (pH 7). The asymmetric aldol reaction could be observed (with 99% yield), but reduced the enantioselectivity (2% ee). This is attributed to presence of high water concentration at the active center that influenced the transition state. Thus, control over the polymer aggregates and aggregation-induced selectivity of aldol product could be realized in response to pH change in aqueous environment. Likewise, Tang et al.^51^ reported L-proline-functionalized pH-responsive block copolymer for asymmetric aldol reaction in water. In this study, a series of block copolymers were employed having L-proline as catalyst moiety, poly(diethylaminoethylmethacrylate) (PDEA) as pH-responsive segment, and methyl polyethylene glycol (mPEG) as hydrophilic moiety (Fig. 2c). Catalytic activity and stereoselectivity of these copolymer catalysts were examined for asymmetric aldol reaction between pNB and cyclohexanone at different pH values (pH = 4.0, pH = 7.0) (Table 1, Entry 10). The result indicated that catalytic activity and stereoselectivity can be affected by the pH value of aqueous solution and the structures of copolymer catalyst assemblies. At acidic pH (pH = 4.0), PDEA fragment of copolymers became hydrophilic due to protonation of amine group and displayed smaller hydrodynamic sizes (~20 nm), poor yield (26%), and selectivity (37% ee). At pH 7.0 (close to the pKa value), copolymer self-assembled to hydrophobic PDEA core and hydrophilic mPEG corona. At this pH (pH 7.0), suitable ratio of hydrophilic to hydrophobic segment of the aggregate effectively attracted substrates to the catalytic sites and avoided large amount of water near the catalytic active center, which can disturb the transition state^52^. The catalytic activity and selectivity of aldol product (75% yield and 89% ee) were increased. However, larger hydrodynamic size (~220 nm) at basic pH (pH = 9.0) indicated the deprotonation of the amine groups leading to more hydrophobic PDEA blocks and less yield of aldol product (59%). Therefore, modulation in chemical conversion and selectivity can be observed by adjusting suitable ratio of hydrophilic to hydrophobic segment of a polymeric material by varying pH.

Besides assemblies in polymeric or supramolecular systems, heterogeneous catalysts have been used for organic transformations due to their site-specific selectivity, minimized metal trace in reaction medium, recyclability, and ease in product separation^53,54^. Among them, core–shell microstructures with inorganic cores and organic shells have been employed for controlled catalysis in aqueous environments^55^. For example, Zhang et al.^56^ employed pH-responsive core–shell microstructures for Suzuki reaction in aqueous media. In this example, Pd nanoparticles (Pd-NPs) were embedded in the shell layer of polystyrene-*co*-poly[2-methacrylic acid 3-bis(carboxymethylamino)-2-hydroxypropyl ester] (PS-*co*-PGMA-IDA). The fabricated structures were stable in basic aqueous solution (pH > 7), and can activate aryl boronic acid to form biaryl product with aryl halides at room temperature (Table 1, Entry 17). High yield of biaryls (>85%) was achieved for hydrophilic substrates, however, poor yield (<30%) was obtained for hydrophobic substrates. Moreover, at lower pH, the core–shell structure was destabilized via protonation of the methacrylate part of the copolymer. This resulted in decrease in reaction rate by restricting access to the Pd particles. Thus, employing Pd-NP fabricated PS-co-PGMA-IDA, the Suzuki reaction in aqueous environment could be switched ‘*on*’ and ‘*off*’ by adjusting the pH of the medium. In this way, accessibility of suitable confined environment for an organic reaction can be controlled by changing pH of the solution. Likewise, core–shell microstructures involving Au-NPs for switchable catalysis was demonstrated by Xiao et al.^57^, where Au-NPs were enveloped by an interpenetrating gel network of methylenebisacrylamide and polyvinylpyrrolidone (PVP) (Table 1, Entry 18). At higher pH (pH > 6), shrinkage of the gel structures occurred due to deprotonation of —COOH group of methacrylate, resulting reduced hydrogen bonding and drainage of water molecules from the core. As a consequence, it closed the diffusion gateways for the reactants for catalytic reduction of 4-nitrophenol with the help of NaBH_4_ (rate of reaction, *K* = 1.0 × 10^−1^ min^−1^ at pH 9.2). In comparison, PVP developed hydrogen bonding with methacrylate groups at lower pH, which permitted the reactants to come in close proximity of the Au-centers, resulting successful reduction reaction (rate of reaction, *K* = 6.1 × 10^−1^ min^−1^ at pH 5.0). Thus, pH-responsive NP-based catalytic systems can be created by combining organic ligands and metal-based NPs^58^.

Catalytic system with Pickering emulsion (PE) provides a distinct platform, which can perform more than traditional catalysis technology^59^. Generally, PE, composed of aqueous-organic biphasic system stabilized by solid particles, can compartmentalize droplets to control the chemical process in response to stimulus, as well as the isolation or protection of incompatible reagents and the sensitive species that can be easily affected by the harsh reaction conditions^60,61^. PE-based systems with control over wettability and interfacial tension of emulsions can provide switchable PE-based catalysts (*vide infra*). pH-responsive PE-based catalytic systems utilize sub-micrometer-sized particles having functional groups that can protonate/deprotonate upon pH change, resulting pH-regulated wettability of the systems. In an example, Yang et al.^62^ reported an in situ preparation method of sub-micrometer size solid catalysts for hydrogenation of styrene via tuning the pH value of solution (Fig. 2d). In this example, hairy silica microspheres were fabricated with a mixture of hydrophilic, pH-sensitive (MeO)_3_SiC_3_H_6_(NHC_2_H_4_)_2_NH_2_ and relatively hydrophobic (MeO)_3_Si(CH_2_)_7_CH_3_ via covalent linkage. Pd-NPs were deposited onto these hairy silica microspheres. This way, interfacially active and pH-responsive solid catalyst was formulated in an organic (toluene)/aqueous biphasic system. The protonation and deprotonation via pH change make the catalyst surface hydrophilic/hydrophobic, resulting switchable emulsion. The system formed an oil-in-water (o/w) emulsion at low pH (pH 3.0 to pH 4.0) due to protonation of triamine-functional group on the surface. In these o/w emulsion, hydrophobic styrene moieties clustered inside the oil droplets. The system availed the favorable conditions for hydrogenation reaction of styrene to ethylbenzene (99% yield) due to the location of the catalyst particles at the interface of the emulsion droplets (Table 1, Entry 20). In contrary, the system is converted to a water-in-oil (w/o) emulsion by increasing the pH value (pH 7.0 to 8.0), where triamine-functional group deprotonated and become hydrophobic. As a result, Pd-NPs of the silica microsphere cannot be accessed by the substrate as it prefers the oil phase, and therefore, the reaction was terminated. The surface of these particles, this way, can be switched between hydrophilic/hydrophobic via protonation or deprotonation by pH change. Moreover, the catalysts can be recycled many times without significant loss of activity. Likewise, pH-switchable catalytic microreactors based on PE were also reported for the reduction of *p*-nitroanisole with NaBH_4_^63,64^.

## Temperature-induced switchable catalytic systems

Temperature is an important stimulus for enzyme catalysis as the rate of enzyme catalytic reactions depends on temperature^65^. Reactions, catalyzed by enzymes, generally stop at higher temperature due to enzyme denaturation. Temperature-responsive artificial switchable catalyst systems have been developed mostly based on temperature-responsive polymers fastened to a catalytic active segment. The temperature-responsive behavior is observed when the temperature is fluctuated around lower critical solution temperature (LCST) of a polymer^66–69^. Depending on the hydrophobic and hydrophilic nature of polymer chain above or below of LCST, the catalytic activity of these polymer materials can be switched ‘*on’* and *‘off’* by obtaining a suitable microenvironment for chemical reactions via adjustment in temperature (*vide infra*). Moreover, employing such polymers as a carrier for metal nanoparticles, nanoreactors have been achieved with the control over catalytic activity just by changing temperature. With respect to temperature-responsiveness, these nanoreactors are broadly classified into two categories—(a) positive temperature-responsive nanoreactors, which deswell at lower temperature, and (b) negative temperature-responsive nanoreactors, which deswell at higher temperature.

**Fig. 3:**
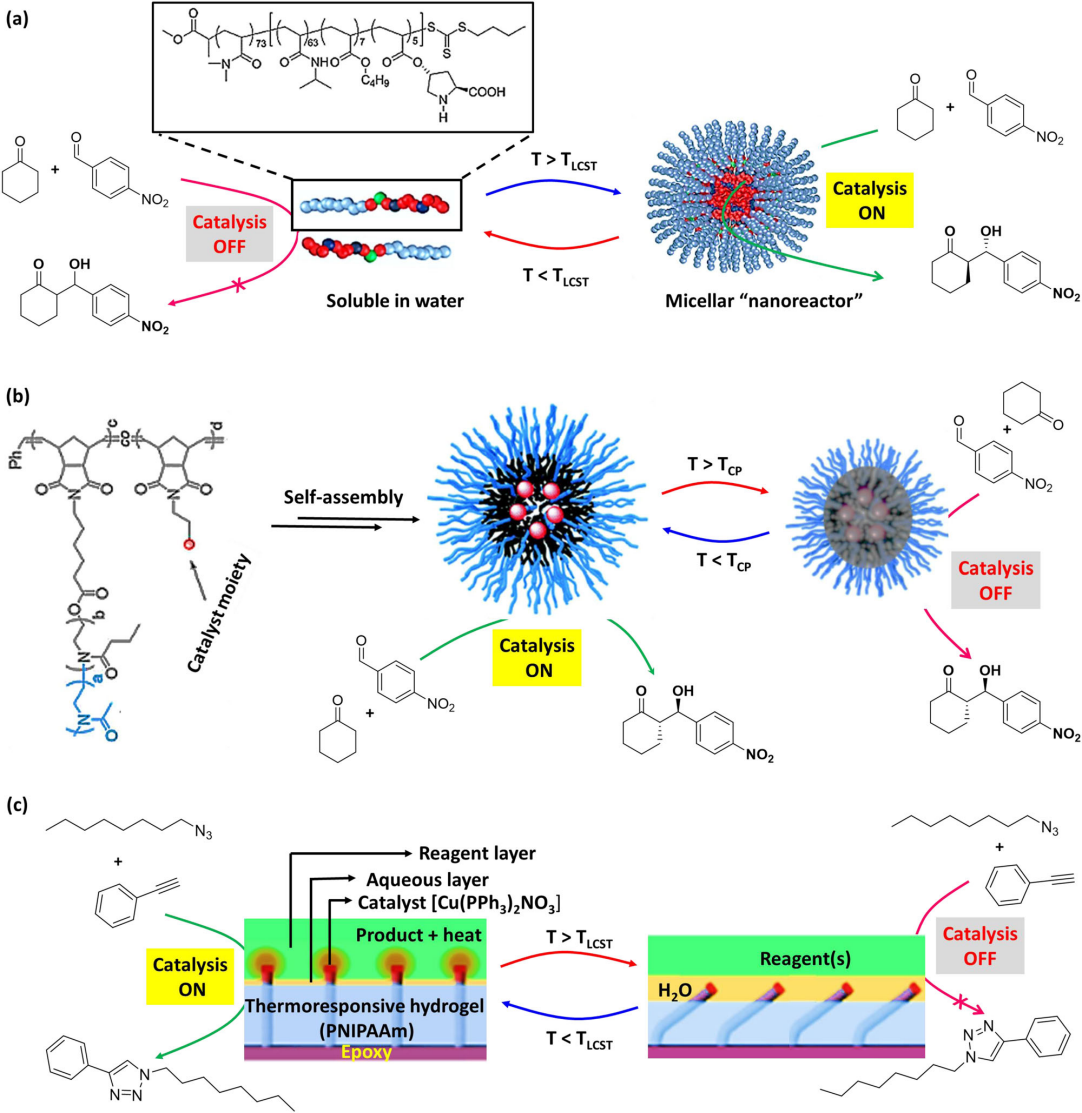
Thermoresponsive polymer-based switchable catalytic systems. **a** Positive thermoresponsive micellar nanoreactor—at lower temperature, the polymer is soluble in water and Aldol reaction is *‘off’* due to the absence of suitable environment (Inset: chemical structure of the polymer). At elevated temperature, micelle is formed and provided suitable environment for L-proline-catalyzed Aldol reaction. Adopted with permission from ref. ^72^, copyright 2013 American Chemical Society. **b** Negative thermoresponsive micellar nanoreactor—at lower temperature, formation of micelle allowed the substrates to access catalyst, whereas at higher temperature polymer chain collapsed to hydrophobic globules, which inhibit the formation of intermediate, resulting poor selectivity. Adopted with permission from ref. ^80^, copyright 2021 American Chemical Society. **c** Self-regulating thermoresponsive catalyst surface—structure tips are coated with catalyst and upright/bent tips corresponding to ‘*on/off*’ catalysis. Below LCST of PNIPAAm, the catalyst tips enter the reagent layer, resulting an exothermic click reaction. Above the LCST, the PNIPAAm contracts and the structures bend, removing the catalyst from the reagent layer and turning ‘*off*’ the reaction. Adopted with permission from ref. ^82^, 2012, Nature Publishing Group, a division of Macmillan Publishers Limited.

Positive thermoresponsive nanoreactors are generally designed in such a way that they show the catalytic activity at elevated temperature. Polymeric micelles have been employed as positive thermoresponsive nanoreactors because of their distinct core–shell structure with confined hydrophobic core domain^70,71^. In an example, Zayas et al.^72^ reported a temperature-induced switchable micellar nanoreactor for asymmetric aldol reaction in water. In the nanoreactor, L-proline was attached with a block copolymer having a permanently hydrophilic block [poly(dimethylacrylamide), PDMA], and a thermoresponsive block [poly(N-isopropylacrylamide) (PNIPAm)] (Fig. 3a). Above the LCST (ranging from 25 to 40 °C) of the polymer, the PNIPAM block became hydrophobic that resulted in micelles of ~15–20 nm in diameter, where the L-proline moiety located within the hydrophobic PNIPAm core. In this way, L-proline moieties in the nanoreactor obtain an ideal hydrophobic environment for catalysis (Table 1, Entry 11). The reactions were efficient at high temperature (50 °C) with excellent yields (95%) and enantioselectivity (96% ee). In contrast, lowering the temperature (below the LCST of the polymer) resulted disassembly of the nanoreactors to water-soluble polymers, and reduction in aldol product formation (53% yield at 25 °C). However, the aldol product was precipitated in ice bath and could be isolated by centrifugation. The aqueous solution of polymer could be reused for next catalytic cycle via reformation of the nanoreactors by increasing the temperature above LCST. Likewise, nanorecators have been realized via immobilizing different organocatalysts, such as imidazole^73^ for hydrolysis of pNPA (Table 1, Entry 2), L-prolineamide^74^ for aldol reaction (Table 1, Entry 12) with thermoresponsive polymers in water.

Catalytic system with microgel can offer an attractive platform as microgels are porous particles consisting of a cross-linked polymeric network, and can form colloidally-stable dispersions in water^75^. By changing temperature, substrates could diffuse through the porous network during swelling in water and can access the catalyst embedded in the network, whereas the molecules would be trapped inside the microstructure during deswelling. Thus, modulation in catalysis can be achieved via temperature-induced swelling/deswelling of microgels. In an example, Tan et al.^76^ reported a temperature-responsive selenium-modified poly(N-vinylcaprolactam) microgel as glutathione peroxidase (GPx) mimic. Diselenide bonds inside the microgel was converted to seleninic acid through oxidation by H_2_O_2_ and used as colloidal catalyst for acrolein oxidation to acrylic acid (Table 1, Entry 22). The catalysis was ‘on’ (yield > 91%) at higher temperature (50 °C), whereas at lower temperature (20 °C) the catalysis was ‘*off*’ and microgel can be removed from the product. However, a substantial decrease in the yield of acrylic acid was reported in consecutive cycles, which is ascribed to higher viscosity of the reaction mixture leading to the deterioration of the diffusion process.

Negative temperature-responsive nanoreactors consisting PNIPAm fragment are often homogeneous below the LCST and can act as an efficient catalyst, whereas above LCST, they form globules, and become a heterogeneous system which shows poor catalytic activity^77–79^. For example, Kuepfert et al.^80^ demonstrated proline-functionalized negative thermoresponsive nanoreactor for aldol reaction in water with efficient catalysis and enantioselectivity (Fig. 3b). In this example, nanoreactors were prepared from proline-functionalized 2-oxazoline-based bottlebrush copolymers, in which the length of 2-oxazoline portion could be adjusted for tunable catalytic activities. Attachment of the proline along the bottlebrush copolymers created a hydrophobic core having the catalyst moiety and a thermoresponsive shell. Upon self-assembly of these bottlebrush copolymers in an aqueous solution, micellar nanoreactor system was generated that catalyze the asymmetric aldol reaction. The micellar nanoreactors exhibit tunable catalytic activity as a function of temperature. The modulation in hydrophobic behavior of the bottlebrush polymer affects the diffusion of substrate molecules to the core. At lower temperature (below the cloud point, T_cp_), proline fragment of the polymer can catalyze the aldol reaction between pNB and cyclohexanone (Table 1, Entry 13) with good conversions (up to 97%) and stereoselectivities (up to 96% ee). In contrary, poor selectivity of the aldol product (12% ee at 80 °C) was obtained at higher temperature. It is proposed that the polymer chain collapse from hydrophilic coils to hydrophobic globules at higher temperature (above T_cp_) resulting enhancement of water diffusion to the catalytic sites that inhibit the formation of stabilized transition state. However, at intermediate temperatures, higher stereoselectivity of the aldol product was observed, which indicated the dependency of water diffusion to the catalytic sites of the micellar nanoreactor is a function of temperature, and selectivity of the aldol product could be modulated via changing temperature. Likewise, thermoresponsive nanoreactor has been realized via immobilizing 4-(dimethylamino) pyridine (DMAP)^81^ for acylation reaction in water (Table 1, Entry 23).

Employing a negative-thermoresponsive hydrogel-based material, He et al.^82^ reported a seminal study about temperature-responsive ‘*on*/*off’* switch for organic reactions. In this example, catalyst moiety was engineered to the tips of an epoxy microstructure via either physical adsorption or chemical covalent attachment process. The resulting assembled system was embedded within temperature-responsive hydrogel PNIPAm, which shows negative temperature-responsive behavior. The system was immersed in a liquid bilayer of aqueous/organic substance (reagent layer) (Fig. 3c). Employing this design, *click* reaction between octylazide and phenylacetylene was performed (Table 1, Entry 24). Below the LCST (32 °C, LCST of PNIPAm), the polymer was in its swollen state and the catalyst-functionalized tips could be found intruding into the reagent layer (octylazide and phenylacetylene). Therefore, the reagents can access Cu(PPh_3_)_2_NO_3_ catalyst, which is on the tip of the microstructure, for *click* reaction. As it is an exothermic reaction, the temperature of the system increased, resulting contraction of the hydrogel above the LCST via expelling water molecules. Thus, the catalyst-bearing microstructure tips were removed from the reagent layer and catalysis was ‘*off’* until the temperature changed to the LCST. Accordingly, self-regulated oscillation of catalysis was generated influencing on the LCST of the hydrogel. Besides the click reaction, Ph_3_CPF_6_ catalyzed decomposition of cumene hydroperoxide (Table 1, Entry 25), H_2_PtCl_6_ catalyzed hydrosilylation reaction between 1-hexene and triethylsilane/diphenylsilane (Table 1, Entry 26) have been successfully performed. By changing the reagent concentration (for controlling the rate of heat generation), the amount of liquid interface, or the microstructure geometry, the temperature oscillations and thereby the catalysis could be finely tuned. These materials are very similar to the living organisms in the sense that they could respond to the changes in their local environment through interconversions of chemical and/or mechanical energy and self-regulating feedback loops^83^.

Besides temperature-responsiveness, the morphology of the PNIPAm segment of a nanostructure has an impact on the activity of the catalyst, which is generally embedded within the hydrophobic core of the nanostructures. In a study, Lu et al.^84^ reported a core–shell (CS) and core−shell-corona (CSC) type nanogels based on L-proline-functionalized hydrophobic core within a thermoresponsive PNIPAm shell. CS nanostructure consists a hydrophobic cross-linked core and a temperature-responsive cross-linked shell, while the CSC morphology consists an additional layer where the polymer chains are less cross-linked. Catalytic dependency of the nanostructures on temperature was examined at three temperatures—at 4 °C where PNIPAm is hydrophilic and solvated, at 25 °C where PNIPAm is somewhat hydrophobic, and at 40 °C (above the LCST of PNIPAm) where it is fully hydrophobic and in a collapsed state. For an asymmetric aldol reaction between pNB and cyclohexanone, it was found that the catalytic activity was enhanced with increasing temperature for CS nanogel structures (40% conversion, 86% ee at 4 °C; 88% conversion, 96% ee at 40 °C), whereas a drop in activity was observed at elevated temperatures with CSC nanostructures (66% conversion, 95% ee at 4 °C; 28% conversion, 97% ee at 40 °C). In comparison, the CSC nanogel showed greater catalytic activity than CS nanogel at low temperature (4 °C), which is attributed to the lower-crosslinking density of the CSC nanostructure, resembling the micellar-type morphology and catalytic activity. With increasing temperature of CS nanogel, the collapse of PNIPAm segment resulted in increase of hydrophobic nature of the core and higher catalytic activity, which is presumably due to increase in substrate uptake and the greater mobility of the substrates within the core. In contrast, for CSC nanogel, the substrates were blocked to access the core presumably due to different-type of collapse for less-cross-linked PNIPAm shell. However, both systems, irrespective of the yields, showed high enantioselectivity for aldol product formation.

Exploiting thermoresponsive behavior of PNIPAm-based materials, metal NPs were entrapped into the polymeric network for the catalysis of various reactions in water^85–89^. In these systems, metal NPs were stabilized by micelle forming amphiphilic block copolymers, and have been employed for a range of catalytic reactions such as hydrogenations, oxidations, reductions, and coupling reactions^90–92^. An interpolymer interaction between the components at lower temperature and disruption of the interpolymer interaction at higher temperature result in this temperature-responsive dynamic nanoreactor system^93–95^. However, colloidal nature of the catalytic sites and the challenges associated with the reversibility of catalyst activity and recycling limit, the use of these type of nanoreactors require further development^96^.

**Fig. 4:**
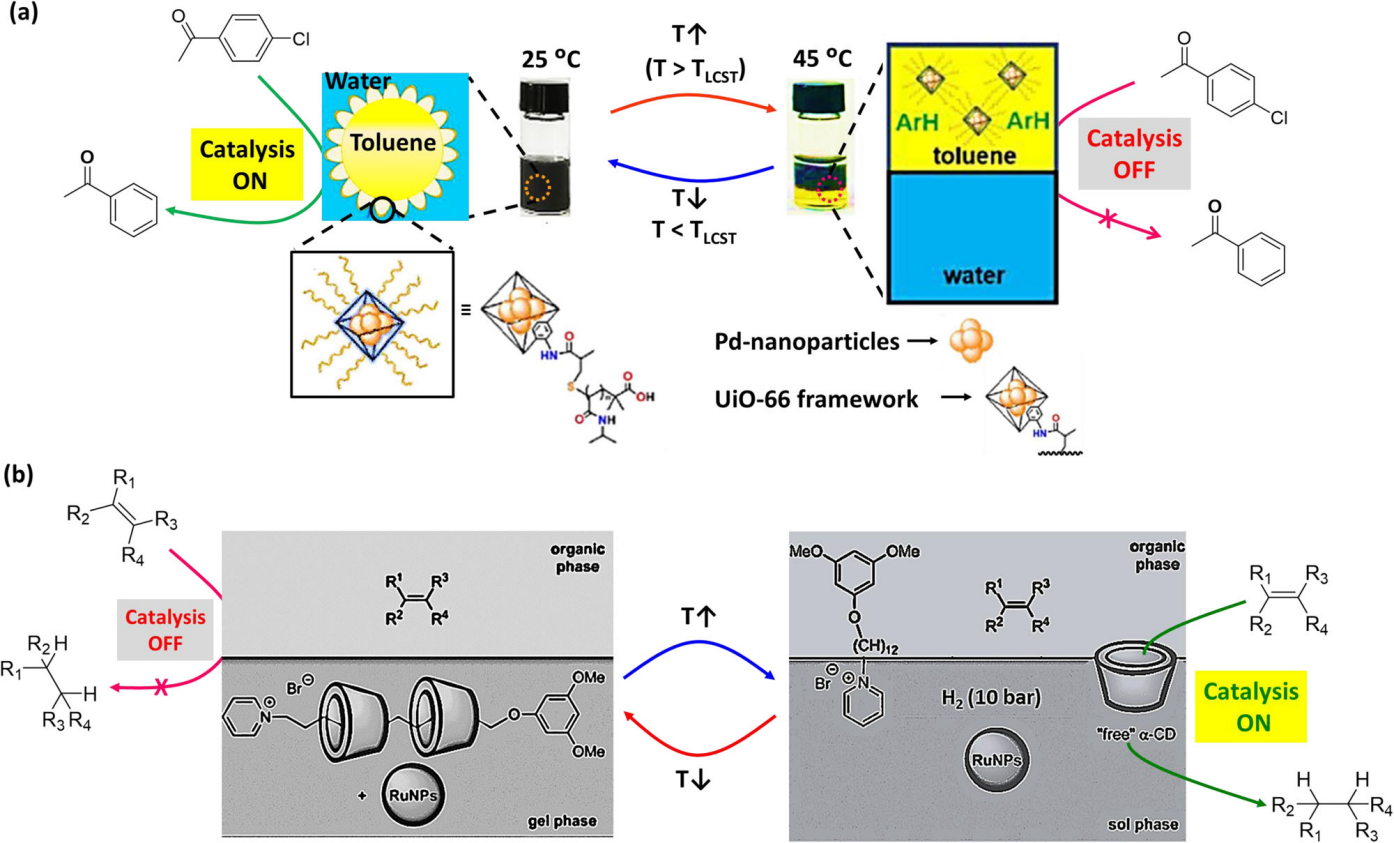
Temperature-switchable nanoparticle-based catalytic systems. **a** PE-based catalytic system—Schematic for MOF-stabilized PE for dehalogenation reactions of chlorobenzene derivatives at 25 °C (Inset: structure of Pd-nanoparticle encapsulated thermoresponsive MOF). By increasing temperature, the system de-emulsified with phase separation and catalytic activity is switched ‘*off*’. Adopted with permission from ref. ^97^, copyright Royal Society of Chemistry. **b** Ru-NPs-based catalytic system—Ru-NPs embedded in the rotaxane-based gel phase, where the alkene cannot access the nanoparticle for catalytic transformation. When temperature has increased, the gel-sol transition occurred, and α-CD could play the role of mass transfer and bring alkenes into Ru-NPs-containing aqueous phase for their conversion into the corresponding alkanes. Adapted from ref. ^105^, copyright 2012 WILEY-VCH Verlag GmbH & Co. KGaA, Weinheim.

Pickering emulsion (PE)-based catalytic system was reported by Dong et al.^97^, which exhibited catalysis of dechlorination reaction as a function of temperature. In this example, PNIPAM was linked with UiO-66 (a nano-sized metal-organic frameworks (MOFs), where UiO-66 is an archetypal MOFs^98^) emulsifier, where Pd nanoparticles (Pd-NPs) were incorporated to create a multicomponent composite emulsifier (Fig. 4a). The system was stabilized with toluene-in-water PE and supported biphasic dechlorination reaction of chlorobenzenes (Table 1, Entry 27). Ammonium formate was used as the reducing agent. At 25 °C (below the LCST of polymer which is 32 °C), hydrophilic PNIPAm brushes stabilized the emulsions, and promoted interfacial catalytic activity of Pd-NPs for dechlorination of chlorobenzene derivatives. In comparison, at an elevated temperature (45 °C, above the LCST of polymer), PE system was demulsified as the PNIPAM brushes become hydrophobic and began to compact on the surface of the MOFs. It resulted organic and aqueous phase separation, which allowed product isolation from organic phase. Reducing temperature back to 25 °C leads to migration of MOFs to aqueous phase, which could be reused after re-homogenization. Thus, PE-based catalytic system could be switched between ‘*on’* and *‘off’* just by varying temperature.

Besides polymer substances, supramolecular hydrogels can act as heterogeneous catalytic system, where reversible covalent self-assembly could be utilized for the development of catalyst systems that can be filtered out after completion of the reaction^99–103^. These systems are interesting due to having large active surface, tunable catalytic activity, and well-ordered arrangement of catalytic groups, which can result enhanced efficiency. However, in most examples, the aldol reaction between pNB and cyclohexanone has been chosen as benchmark reaction^104^. Metal nanoparticles can be embedded into supramolecular hydrogel structure and catalytic activity can be switched employing temperature stimulus. In an example, Léger et al.^105^ developed cyclodextrin-based thermoresponsive hydrogel incorporating catalytically active ruthenium nanoparticles (Ru-NPs) for hydrogenation reactions (Fig. 4b). In this example, Ru-NPs were embedded into a supramolecular hydrogel matrix consisting N-alkylpyridinium amphiphile and α-cyclodextrin (α-CD). In water, the amphiphile and α-CD self-assembled to [3]pseudorotaxane, where the amphiphilic character of pyridinium-derivative was masked via alkyl chain inclusion into the cavities of α-CD, resulting in the formation of a hydrogel. At high temperature, the association constant between α-CD and the amphiphile become sufficiently low, resulting free pyridinium-derivative and α-CD. Therefore, as a supramolecular carrier, α-CD influenced the mass transfer between NPs-containing aqueous phase and substrate-containing organic phase. Above the sol–gel transition temperature (39 °C), the system transformed to sol phase, where the catalytic hydrogenation of terminal alkenes took place (Table 1, Entry 28). The Ru-NPs, stabilized in the hydrogel network, demonstrated a pronounced catalytic activity at 50 °C. In contrast, after cooling the temperature to ambient condition, the system spontaneously returned to the gel state, and showed no catalytic activity. However, the hydrogenated products and catalyst could be easily separated at lower temperature. It is worth noting that the dispersion of Ru-NPs in the hydrogel network remained intact for reuse.

Reversible nature of supramolecular gels by changing the temperature can allow a reversible sol–gel transition and thereby the modulation in catalysis. This type of tunable catalyst may find interesting applications, especially if different stimuli such as light, sound, magnetic field can be used to regulate hydrogel formation and subsequent catalysis.

## Light-induced switchable catalytic systems

Light stimulus is advantageous over other stimuli due to its noninvasive nature, easy modulation via monitoring the source, and its precise physicochemical control over reaction^106^. Photoresponsive processes found in nature illustrate the use of light irradiation for initiating and regulating complex molecular and biochemical processes. Different approaches can be employed in artificial photoresponsive systems^107–109^, including photocatalysis, photoswitchable catalysis. In photocatalysis, upon light irradiation, an inactive precatalyst provides the catalytically active photoexcited state that reacts with a substrate, whereas a catalytically active species undergoes a reversible photochemical transformation in photoswitchable catalysis resulting the change in its intrinsic catalytic properties^110^. Photoswitchable catalysis offers distinct advantages over photocatalysis as the former provides an extra handle for controlling the catalyst activity. These photoswitchable catalytic systems are generally obtained via incorporating photoactive units in the molecular architecture. Electronic effect, steric effect, cooperative effect are the principal type of effects utilized to achieve control over these systems. In the following, we describe light-triggered switchable catalyst systems for organic transformations in aqueous environments.

**Fig. 5:**
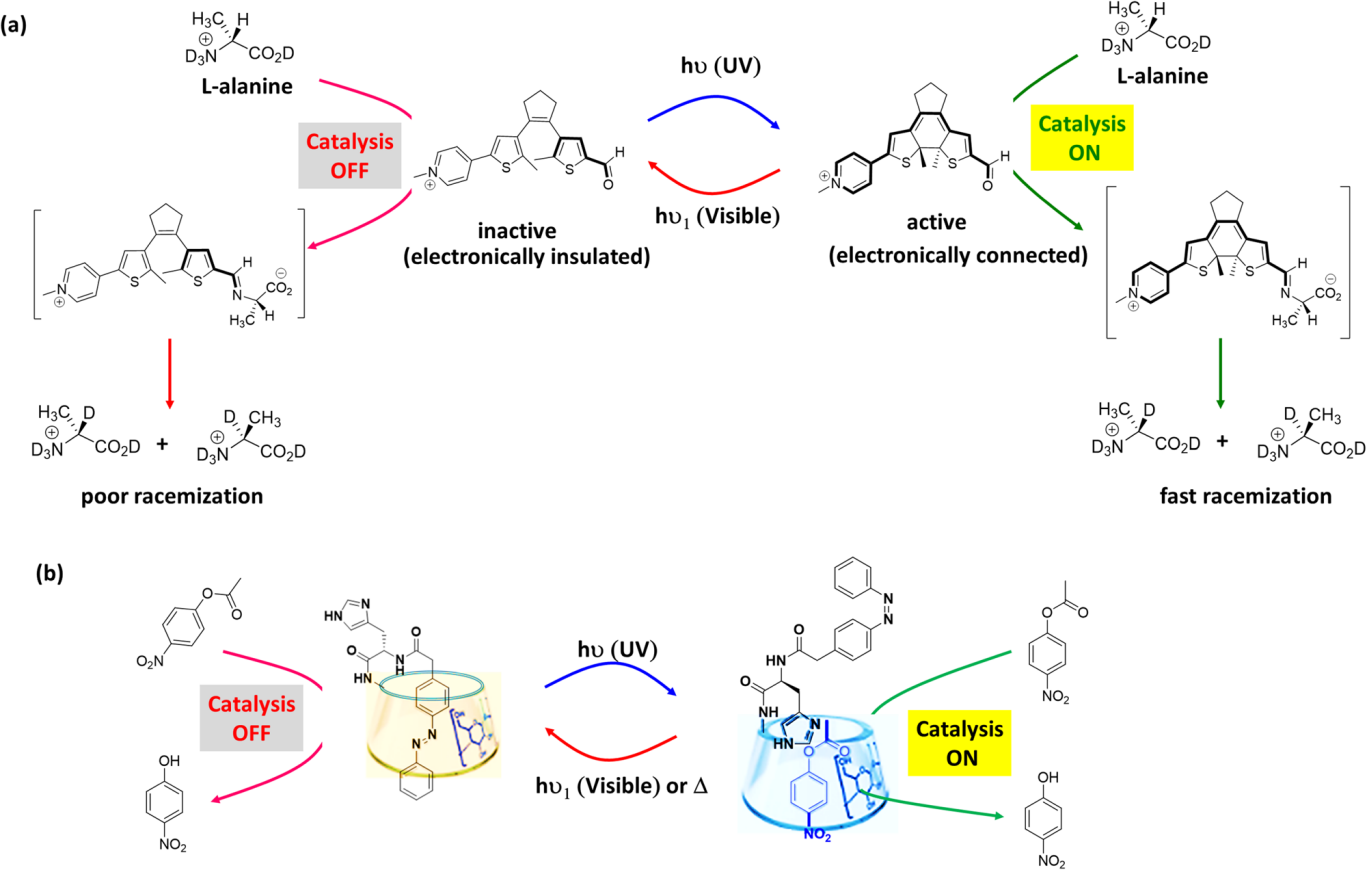
Light-switchable catalytic systems. **a** Photoresponsive PLP mimic—Light-induced isomerization of dithienylethene structure between its “inactive” ring-opened and “active” ring-closed indicating whether the pyridinium and aldehyde are insulated or connected to each other for providing aldimine from reaction of aldehyde derivative and L-alanine. **b** β-cyclodextrin-based catalyst system—*trans*-azobenzene makes inclusion complex with the cavity of β-CD, resulting unavailability of catalytic site for the hydrolysis reaction. Light irradiation results *cis*-azobenzene that excluded from the cavity of β-CD allowing ester molecules to access the catalytic site in the cavity, which resulted faster hydrolysis. Adopted with permission from ref. ^115^, copyright 2001 WILEY-VCH Verlag GmbH, Weinheim, Fed. Rep. of Germany.

Drawing inspiration from light-controlled biochemical process, controlling the catalytic activity via electronic modulation can be achieved. For example, pyridoxal-5-phosphate (PLP), which is biologically active form of vitamin B6, is a versatile enzyme cofactor used by nature for several biosynthetic procedure^111^. The action of PLP depends on the electronic connection between aldehyde group and pyridinium group. The pyridinium group in the aldimine (formed by the condensation of an amino acid and PLP) enhances the acidity of the α-hydrogen by stabilizing the conjugate base through contributions from the quinonoid structure. A photoresponsive mimic of PLP was demonstrated by Wilson et al.^112^, where diarylethene photoswitch was replaced in the PLP core ring. In the ring-opened isomer, the pyridinium and aldehyde functional groups were electronically insulated, which precluded catalytic activity (Fig. 5a). On the other hand, irradiation of UV light (*λ* = 365 nm) provided fully conjugated ring-closed form, where aldehyde and pyridinium groups are electronically connected. Therefore, the ring-closed form provides the stabilized quinonoid structure upon condensation with L-alanine (an amino acid), and deprotonation of the aldimine α-hydrogen. Thus, treatment of L-alanine with ring-opened isomer resulted in poor racemization reaction (3% yield), whereas an increase in the rate of racemization was observed upon UV irradiation to ring-closed form (Table 1, Entry 29). The ability of light-responsive PLP mimic, as a controllable catalyst, was demonstrated employing hydrogen–deuterium exchange experiments in a mixture of D_2_O and CD_3_CO_2_D. Furthermore, reversible switching the catalytic activity between its opened-form (catalysis ‘*off*’) and its closed-form (catalysis ‘*on*’) was demonstrated through alternate exposure to UV (*λ* = 365 nm) and visible light (*λ* > 490 nm).

The *trans* → *cis* isomerization of an azobenzene or stilbene moiety can be used for catalytic purpose in aqueous media enabling steric modulation via large geometrical change due to the isomerization process. In these systems, the active site of the catalyst would be inaccessible to the reactants by a group such as host–guest binding, bulky functional group to shield the catalytic sites. Light-induced isomerization would result the geometrical change and unblocking of catalytic site to the reactants. In an early example, Ueno et al.^113^ exploited host–guest interactions to modulate the catalytic activity, where β-cyclodextrin (β-CD) acts as host and 4-carboxyazobenzene acts as guest. The azobenzene derivative changes the rate of the hydrolysis of pNPA in tris buffer medium (pH 8.7) (Table 1, Entry 3). Complexation between *trans*-azobenzene moiety and β-CD effectively block one end of β-CD, resulting inaccessible β-CD for the ester substrate, and thereby inhibition of hydrolysis (rate constant, *K* = 1.17 × 10^−4^ S^−1^ for uncatalyzed reaction). However, upon irradiation of UV light (*λ* = 365 nm), *tans* → *cis* isomerization led to exclusion of *cis*-azobenzene moiety from β-CD, which resulted inclusion of pNPA in the cavity of β-CD. This facilitates attack of one of the peripheral hydroxyl groups of β-CD on the ester moiety. Therefore, enhancement in the rate of hydrolysis (rate constant, *K* = 1.56 × 10^−4^ S^−1^ for catalyzed reaction) was observed. Moreover, *cis*-azobenzene moiety can isomerize back to *trans-*azobenzene moiety utilizing thermal energy or irradiation of visible light, resulting the inhibition in ester hydrolysis rate. Likewise, photoswitchable catalysts were developed based on covalently attached azobenzene moieties to the lower rim of β-CD^114^. Photocontrolled imidazole catalyzed hydrolysis of esters in phosphate buffer (pH 7.2) was reported by Lee et al.^115^ employing a functionalized β-CD with an azobenzene unit and a histidine residue (Table 1, Entry 4). In this example, an imidazole group was attached to an azobenzene-based pendant and the catalytic site is unavailable for the ester molecules as *trans*-azobenzene effectively makes inclusion complex with host cavity of β-CD (Fig. 5b). Conversely, light irradiation results *cis*-azobenzene, which could not be included in the cavity of β-CD and hence the binding site is available for the insertion of both the ester molecule and imidazole moiety, resulted faster hydrolysis of various ester molecules, namely pNPA (kinetic efficiency = 0.27 s^−1^ M^−1^), Boc-L-alanine-*p*-nitrophenyl ester (kinetic efficiency = 1.0 s^−1^ M^−1^) and Boc-D-alanine-*p*-nitrophenyl ester (kinetic efficiency = 0.96 s^−1^ M^−1^). Furthermore, the azobenzene moieties were functionalized to gold nanoparticles (Au-NPs) that bind Zn^+2^ coordinated β-CD dimer as a heterogeneous photoswitchable system to modulate ester hydrolyses^116^. Upon complexation, the NPs aggregated and showed poor catalytic activity (rate constant, *k* = 54.1 × 10^−8^ M min^−1^ for *trans*-isomer). UV light irradiation led to *tans* → *cis* isomerization, resulting exclusion of *cis*-azobenzene moiety from β-CD. NPs deaggregation and enhancement in ester hydrolysis rate were observed (rate constant, *k* = 132 × 10^−8^ M min^−1^ for *cis*-isomer). These, light-induced switching of catalytic activity is an excellent example with respect to artificial molecular switching systems in aqueous medium.

**Fig. 6:**
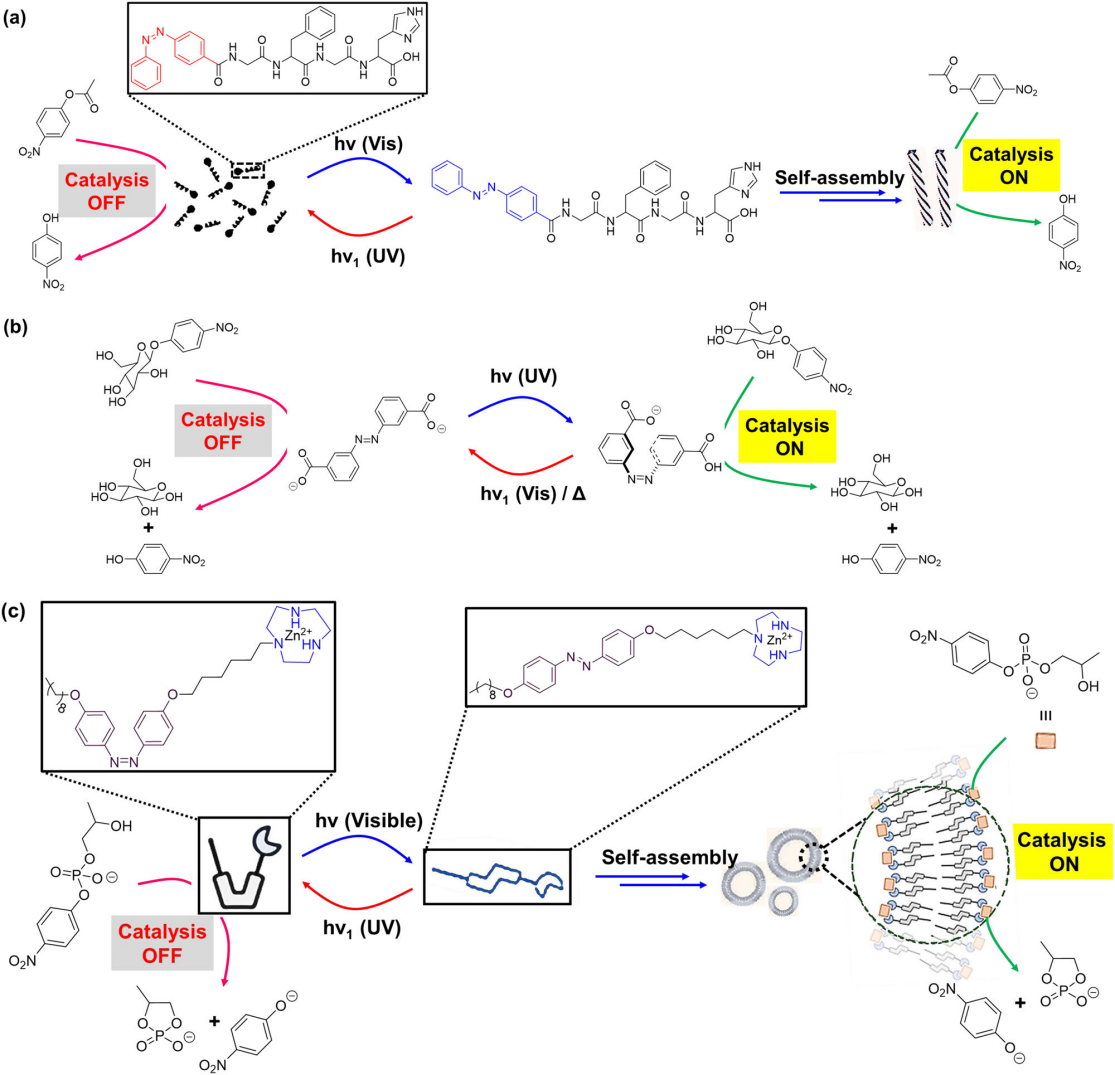
Light-switchable catalytic systems based on cooperativity effect. **a** Peptide-based artificial hydrolase—azobenzene-functionalized peptide molecules can self-assemble into nanofibers, where basicity of imidazole is enhanced for hydrolase-like catalytic activity on pNPA. The activity can be switched ‘*off’* under UV light irradiation via *trans* → *cis* photo-isomerization of the azobenzene group, leading to the disassembly of nanofibers. Adopted with permission from ref. ^117^, copyright 2018 Royal Society of Chemistry. **b** Photoswitchable glycosidase mimic – deprotonation of the carboxylic acid groups from *cis*-isomer of azobenzene-functionalized dicarboxylic acid occurs in a stepwise fashion, whereas deprotonation for *trans*-isomer occurs simultaneously. The monoanionic form of the *cis*-isomer can act as a glycosidase mimic that proceeds through a general acid-base catalytic mechanism for the hydrolysis of 4-nitrophenyl-β-D–glucopyranoside. Catalysis via the cooperative mechanism is absent for *trans*-isomer. **c** Photoswitchable self-assembled catalytic system—the catalysis can be switched between the ‘*on*’ and ‘*off*’ states by light irradiation. The *trans*-isomer of an amphiphile self-assembles into vesicular structures, which show cooperative catalysis for transphosphorylation reaction (Inset: *cis*- and *trans*-isomer of the amphiphile). UV light irradiation provides the *cis*-isomer, resulting disassembly and switching ‘*off*’ the catalysis. Adopted with permission from ref. ^123^, copyright 2019 Wiley-VCH Verlag GmbH & Co. KGaA, Weinheim.

Regulation of enzyme-like catalytic activity could be achieved in artificial systems by introducing flexible domains in photoswitchable systems for enabling preorganization and induced fit. In an example, Zhao et al.^117^ reported an artificial photoswitchable hydrolase activity by incorporating an azobenzene group to peptide sequence Gly-Phe-Gly-His (GFGH) (Fig. 6a). The peptide can self-assembled into nanofibers, by which basicity of the imidazole functional group of histidine was enhanced based on the cooperative effects. The hydrophobic microenvironment in the supramolecular assemblies was suitable for the hydrolase-like catalytic activity on pNPA in PBS buffer (pH 7.4) (kinetic efficiency = 13.95 × 10^−3^ min^−1^ mM^−1^ for *trans*-isomer) (Table 1, Entry 5). Upon UV irradiation, the azobenzene moiety isomerizes to *trans* → *cis* conformation, resulting break down of β-sheet nanofibers to random coils and consequent reduction in catalytic efficiency (kinetic efficiency = 11.08 × 10^−3^ min^−1^ mM^−1^ for *cis*-isomer). This way, the activity of the hydrolase mimic can be switched reversibly employing UV and visible light. However, repeated cycles of activation and deactivation via light irradiation appeared to disturb the supramolecular architecture, and reduction in catalytic ability.

Due to light-induced isomerization process, a change in the distance or orientation of catalytic sites of switchable catalyst can modulate the rate of chemical process^118^. Artificial photoresponsive glycosidase mimic was reported by Samanta et al.^119^ employing a photoresponsive bifunctional organocatalyst based on the cooperative effects of azobenzene-functionalized biscarboxylic acid (Fig. 6b). Glycosidase enzyme is responsible for hydrolysis of glycosidic bond between two glucose residues of biopolymers^120^. One of the carboxyl groups of glycosidases functions as general acid catalyst, whereas other carboxylic group acts as a nucleophile, forming a covalent glycosyl-enzyme intermediate. In the glycosidase mimic, the carboxylic acid groups of *trans*-azobenzene isomer ionize at roughly same pH, whereas deprotonation of *cis*-azobenzene isomer, which is obtained via 366 nm light irradiation, occurs in a stepwise fashion and it exists as the monoanionic species between pH 4.7 and 6.5. An increase in the reaction rate of six order was observed with respect to background reaction at pH 5.8 for the hydrolysis of 4-nitrophenyl-β-D-glycopyranoside (kinetic efficiency = 9.2 × 10^2^ mM^−1^ min^−1^) (Table 1, Entry 30), which is similar in terms of functionality as the active form of a glycosidase. Moreover, the catalytic activity could be switched ‘*on/off’* via altering exposure to UV/visible light.

The carbonic anhydrase (CA) enzymes catalyze the reversible hydration or dehydration of CO_2_/HCO_3_^−^. It consists a distorted tetrahedral Zn(II) complex coordinated to imidazole nitrogen atoms of histidine residues and a water molecule^121^. Photoresponsive CA enzyme mimic was reported by Saha et al.^122^ for pNPA hydrolysis, where the CA-activity can be turned ‘*on/off*’ reversibly by irradiation of light in PBS Buffer (pH = 7.6) (Table 1, Entry 7). This example employed a photoregulated CA mimic with Zn(II) complex of two imidazoles appended azobenzene system. The *cis*-azobenzene isomer formed a 1:1 complex with Zn(II) ions that showed efficiency as an active-site mimic of the CA enzyme (kinetic efficiency = 0.24 mM^−1^ min^−1^). In contrast, the *trans*-isomer provided a polymeric —(Zn(II)-*trans*-)— network, which showed poor efficiency towards hydrolysis reaction of pNPA.

Artificial catalysts have the potential for controlling catalysis within self-assembled structures via the combination of cooperativity and self-assembled structure formation. In an example, Ren et al. ^123^ reported self-assembly of azobenzene-based amphiphilic molecules for creating catalytic pockets utilizing intermolecular cooperative effect that can be switched between ‘*on/off’* states via light irradiation. In this example, azobenzene-functionalized 1,4,7-triaza-cyclononane (TACN) derivative self-assembled into vesicular structures in a HEPES buffer solution (pH 7) (Fig. 6c). The molecular assembly can effectively catalyze the transphosphorylation reaction (rate = ~0.5 × 10^−8^ mol s^−1^ for *trans*-isomer) of 2-hydroxypropyl-4-nitrophenylphosphate (HPNPP), which is a model substrate for RNA hydrolysis (Table 1, Entry 31). UV light irradiation (*λ* = 365 nm) converts *trans* → *cis*-isomer, resulting disassembled states and poor catalytic activity (rate = ~0.17 × 10^−8^ mol s^−1^ for *cis*-isomer). Moreover, conversion of *cis* → *trans*-isomer and thereby the assembled state could be obtained via light irradiation from a white LED lamp. Thus, light irradiation within the amphiphilic molecule can allow switching between assembled and disassembled states, thereby modulating the rate of chemical transformations. Likewise, the polarity change in 4-(phenylazo)-benzoate derivative due to light irradiated *trans* ↔ *cis* isomerization was utilized by Neri et al.^124^ for controlling the binding affinity to Au-NPs and thereby their catalytic activity. In this example, the Au-NPs were functionalized with a monolayer of alkyl thiol terminated with aTACN—Zn^2+^ headgroup, which can catalyze the transphosphorylation of HPNPP in aqueous buffer (pH 7.0) (Table 1, Entry 32). Light-sensitive 4-(phenylazo)-benzoate was used as a cofactor to regulate the catalytic activity of the nanosystem. The catalytic activity was downregulated with the *trans*-azobenzene derivative which was attributed to its higher binding affinity to the catalyst. In contrast, the *cis*-azobenzene derivative disfavored its hydrophobic binding with the apolar part of the Au-NP-attached monolayer due to the increased polarity, and increased the reaction rate up to 1.5-fold. Although several operational limitations of this system were reported, but reversible formation of cooperative catalysts within a dynamic self-assembled system is a promising new tool for the design of complex artificial systems in aqueous environment.

**Fig. 7:**
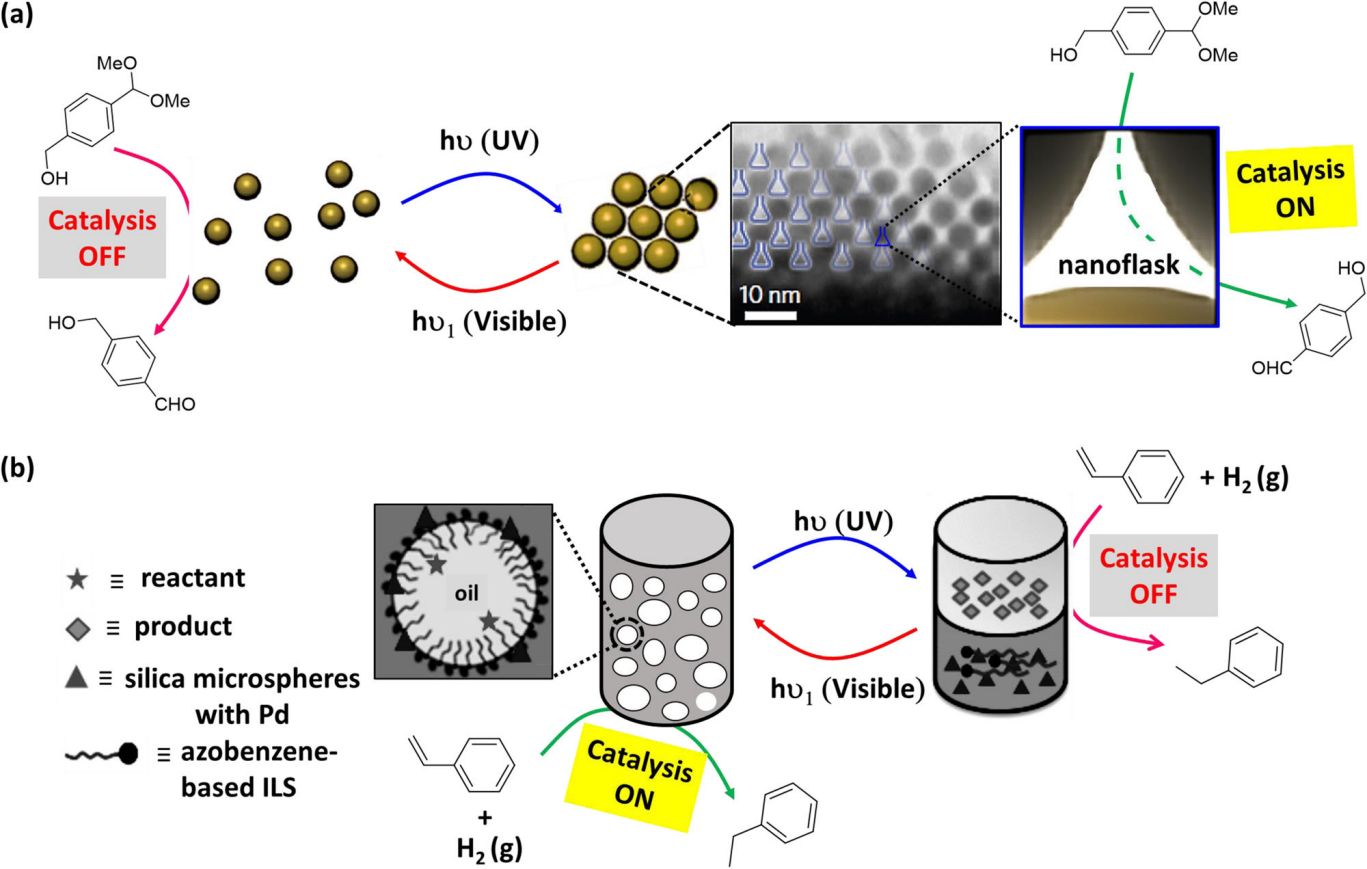
Light-switchable nanoparticle-based catalytic systems. **a** Light-triggered dynamic formation of ‘nanoflasks’ based on azobenzene-functionalized Au-NPs—upon UV irradiation, *cis*-isomer aggregated to provide the nanoflask, which provide suitable atmosphere for acetal hydrolysis. Visible-light-triggered *trans*-isomer disintegrated the nanoflask and consequently switching ‘*off*’ the catalysis. Adopted with permission from ref. ^127^, copyright 2015, Nature Publishing Group. **b** Light-switchable PE-based system—*trans*-azobenzene-based catalytic system efficiently catalyzes hydrogenation reaction at ambient condition. UV irradiation results phase separation and switching *‘off’* the catalysis, whereas visible light irradiation and homogenization results emulsification for catalytic reaction. Adopted with permission from ref. ^128^, copyright 2020 Wiley-VCH GmbH.

Self-aggregation of NPs could provide suitable hydrophobic inert environment amidst surrounding aqueous medium^125,126^. In a seminal work, Zhao et al.^127^ described novel class of synthetic confined environments, whose formation and disassembly are governed by light irradiation. In this example, azobenzene-functionalized Au-NPs were used for reversibly creating and destroying confined environments (‘nanoflasks’), where trapped molecules can undergo chemical reactions with increased rates (Fig. 7a). Upon UV light irradiation, *trans* → *cis* isomerization of azobenzene moiety paves the pathway for aggregation of the NPs and formation of nanoflasks where reaction rate increases. Afterwards, exposure of visible light results *cis* → *trans* isomerization and subsequent disintegration of the ‘nanoflasks’ assuring product recovery at the desired yield. Acid-catalyzed hydrolysis of acetal to aldehyde in water-saturated toluene in presence of functionalized Au-NPs was investigated (Table 1, Entry 35). The reaction was observed to have proceeded at much higher rates as compared to that in absence of the aggregates or UV irradiation.

Azobenzene photoswitches were employed in regulating the stabilization of Pickering emulsions (PEs) and catalysis in such systems. In an example, Li et al.^128^ reported light-induced emulsification and demulsification of ionic liquid surfactants for catalytic hydrogenation of styrene (Fig. 7b). The PE was stabilized by silica microspheres functionalized with surface-loaded Pd and water-soluble azobenzene-based ionic liquid surfactant (ILS). Catalytic system Pd/SiO_2_/ILS was found to actively participate in catalytic hydrogenation of styrene with excellent conversion at ambient condition (Table 1, Entry 21). Upon UV light irradiation, *trans*→*cis* isomerization of azobenzene moiety resulted increase in hydrophilicity of ILS and decrease of surface activity, consequently destabilization of emulsion. A complete phase separation of aqueous phase and organic phase provided poor rate of catalytic reaction, but ease of product separation. Moreover, irradiation of visible light to the system, followed by homogenization, reformed the stable PE for reuse. Thus, PE can be used as a microreactor, which allowed controlled catalytic reaction, product separation, and catalyst recycling just via alternate exposure to UV and visible-light irradiation.

Light-induced reversible change in wettability of TiO_2_ surfaces has been utilized in photoswitchable heterogeneous catalysis. In presence of UV light, water molecule (present in moisture) can coordinate with the titanium atom of a TiO_2_ surface, which resulted increase in number of –OH groups, and the hydrogen bonding ability of the surface^129^. Thus, the number of –OH groups of a surface could be modulated via UV light irradiation and keeping in the dark. Various hydrogen bond catalyzed organic reactions were observed on these surfaces, including epoxide ring-openings (33–38% yield under UV irradiation, whereas 0–9% yield in dark), cycloadditions (54–76% yield under UV irradiation, whereas 30–34% yield in dark), and C–C forming reactions (64% yield under UV irradiation, whereas 18% yield in dark). The reversible wettability was also employed for switching the rate of aldol reaction between benzaldehyde and acetophenone. Thus, a broad range of C–C bond-forming chemical reactions could be modulated by using heterogeneous catalysts, such as TiO_2_ via light irradiation.

## Small molecule-induced switchable catalytic systems

Self-assembly of amphiphilic molecules in water can provide nano- or microstructures such as micelles, vesicles, and emulsions, which can be used as nanoreactors by offering suitable reaction space from the environment^130–133^. However, controlling and regulating these nanoreactors dynamically and reversibly employing small molecules is a great challenge.

**Fig. 8:**
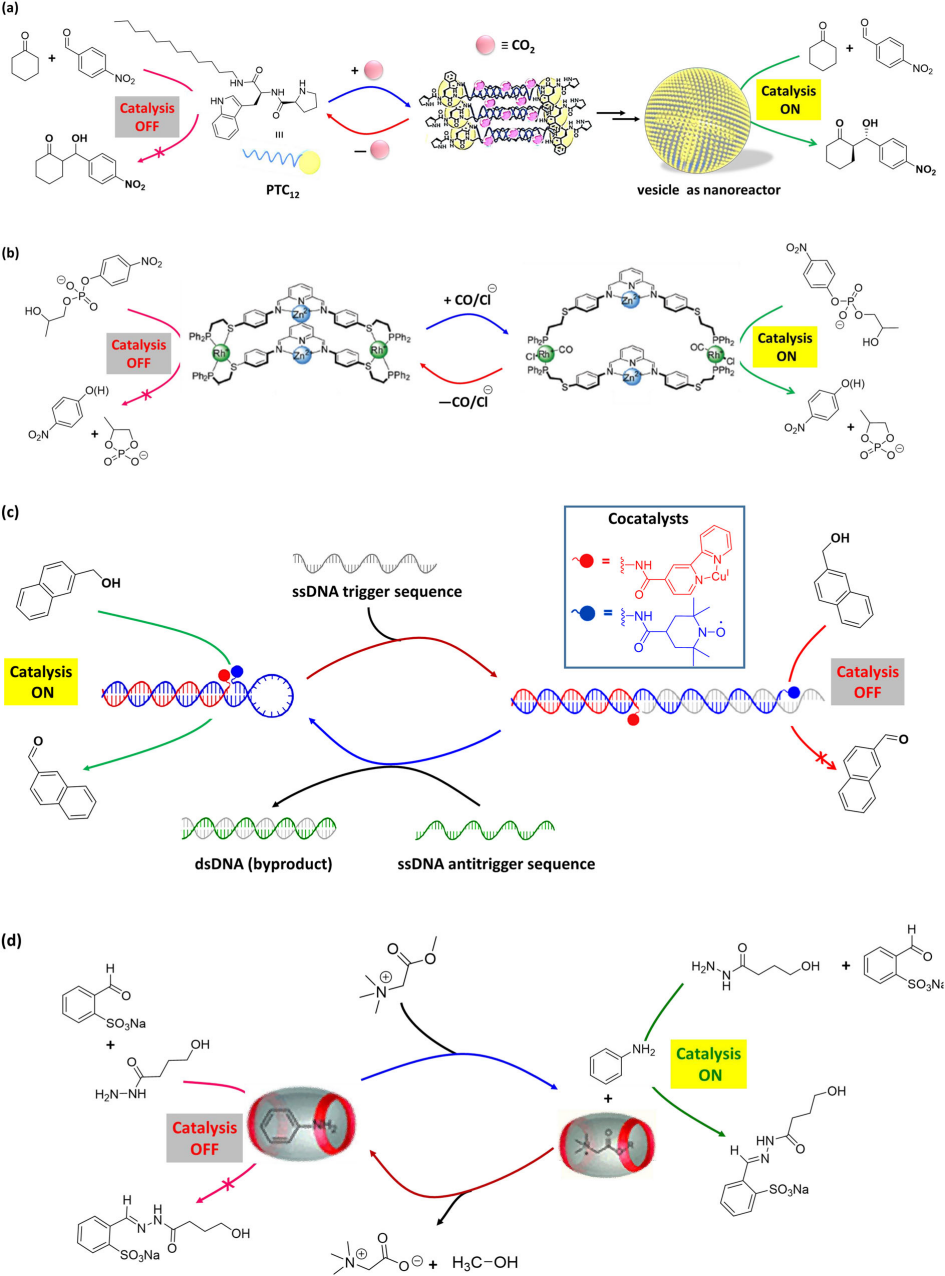
Small molecule-responsive switchable catalytic systems. **a** CO_2_-responsive switchable system—amphiphilic molecule, PTC_12_ is insoluble in water and catalytic aldol reaction is switched ‘*off*’. In presence of CO_2_, PTC_12_ self-assembles to vesicle nanoreactor, where catalysis is turned ‘*on*’ for efficient aldol reaction. Adopted with permission from the ref. ^134^, copyright 2013 WILEY-VCH Verlag GmbH & Co. KGaA, Weinheim. **b** Small molecule/ion-induced supramolecular allosteric catalyst—use of small molecule regulators (CO and Cl^−^) change the size of the macrocycle, the accessibility to the active binuclear Zn site, and thereby the catalysis of HPNP hydrolysis reaction. Adopted with permission from the ref. ^140^, copyright 2007, American Chemical Society. **c** DNA duplex-scaffold functionalized with Cu-bpy and TEMPO for switchable catalysis—the distance between the two cocatalysts in DNA architecture can be altered upon introduction of ssDNA strand (Inset: structures of Cu(I)-bpy and TEMPO cocatalysts). Presence of an ssDNA trigger sequence, the structure holds the cocatalysts apart and turning ‘*off*’ the catalysis. The original catalyst conformation is restored upon addition of an ssDNA antitrigger strand that catalyze the oxidation of 2-naphthalenemethanol in borate buffer (pH 9.5) environment. Adopted with permission from ref. ^143^, copyright 2021, American Chemical Society. **d** Chemical fuel-induced transient availability of catalyst for a chemical reaction—glycine betaine methyl ester competes for CB[7] binding with aniline, and their hydrolysis controls the release of aniline from CB[7] for hydrazone formation reaction. Glycine betaine methyl ester binds with CB[7], allowing the release of aniline and turning ‘*on*’ the catalysis. Rebinding of aniline in the cavity of CB[7] due to decay of ester molecules switch ‘*off*’ the catalysis. Adopted with permission from ref. ^165^, copyright 2022 American Chemical Society.

Compressed CO_2_ can trigger the formation of nanoreactors such as nanoemulsion. Reversibility between micelle-to-vesicle transitions can be controlled by regulating the pressure of CO_2_ and eventually the activity and selectivity of a catalyst. Qin et al.^134^ employed a proline-based amphiphilic molecule (PTC_12_, Fig. 8a) having a long alkyl chain at the C-terminus to form vesicle under compressed CO_2_. The peptide-based amphiphilic PTC_12_ was insoluble in water. However, upon introduction of compressed CO_2_, PTC_12_ formed bicarbonate salt that increases the hydrophilicity of PTC_12_ and consequently facilitates the formation of vesicular assembly in water. The hydrogen bonds between amide functionality among head groups reduced the surface area per surfactant headgroup, resulting the formation of vesicles. Besides, compressed CO_2_ regulated the size of the vesicles via inserting CO_2_ into the hydrophobic area of the packed amphiphiles. The vesicle structures can catalyze a direct aldol reaction with high selectivity (99% yield, 93% ee) (Table 1, Entry 14). Moreover, the nanostructures were regulated by the presence of CO_2_, providing a dynamic regulation of the system where the catalyst activity and selectivity can be controlled. The amphiphilic proline derivative could be reused to form the vesicles for catalyzing the reaction. Such process could be done several times just via alternate exposure of CO_2_ to the system or by removing CO_2_ from the system. This is an interesting example that bridge the gap between random and unstable catalytic emulsion systems and the ordered and stable catalytic hydrogels via employing small molecule such as CO_2_.

Nature has evolved suitable pathways for controlling the hydrolysis of phosphate diesters in important biomolecules such as DNA and RNA. Many of the enzymes, such as P1 nuclease, DNA polymerase I, phospholipase C, and alkaline phosphatase, depend on the synergistic action of two metal centers (typically Zn^2+^) for promoting the hydrolytic cleavage of the phosphate diester bonds^135,136^. In artificial systems, allosteric control over switchable catalysis has emerged in recent years via the use of (nano)mechanical reorganizations^137–139^. In an example, Yoon et al.^140^ described a switchable catalytic system having Zn(II)-pyridine-bisimine catalytic motifs. The system can undergo reversible switching triggered by the addition or removal of chloride ion (Cl^−^) and carbon monoxide (CO) in a pseudo-aqueous solution (Table 1, Entry 33). The hydrolysis of HPNP (Fig. 8b) could be turned ‘*on*/*off*’ in the networked system employing a metalation/demetalation protocol, where weak-link approach (WLA)^141^ is exploited. No conversion was observed in the closed state of the catalyst (catalysis ‘*off*’), while the open state led to full conversion (catalysis ‘*on*’). The absence of activity in the ‘*off*’ state was ascribed to the short distance between Zn atoms that inhibited the bimetallic intramolecular reaction. On the other hand, the presence of monodentate ligands (CO or Cl^−^) at rhodium (Rh) centers allowed a switch to a flexible semi-open form as ‘*on*’ state from a rigid-closed form as ‘*off*’ state. The semi-open structure can be switched back to the rigid-closed form just by removal of these monodentate ligands.

Artificial synergistic catalysis, combining two cocatalysts in close proximity, work in concert for carrying out a reaction, which could not be possible emloying either cocatalyst alone^142^. Recently, Pimentel et al.^143^ demonstrated DNA-based scaffold for switchable synergistic catalysis through tuning the geometric relationship between the cocatalysts (Cu and 2,2,6,6-tetramethylpiperidine-1-oxyl [TEMPO]) for oxidation of naphthalenemethanol in aqueous borate buffer (pH 9.5) medium (1/1 borate buffer/acetonitrile) (Table 1, Entry 36). In this example, DNA-cocatalyst conjugates were prepared via bioconjugation of two cocatalysts (Cu and TEMPO) to the end of a DNA helix (Fig. 8c). DNA duplex was obtained via self-assembly of DNA-cocatalyst conjugates that shows much better catalytic activity compared to unscaffolded cocatalysts (without DNA duplex). This is due to the precise placement of the cocatalysts in the DNA duplex. Moreover, the DNA backbone allowed the switching of catalytic activity exploiting a strand-displacement approach. A dynamic DNA structure was created just by incorporating a hairpin motif into the sequence of one of the cocatalyst conjugates. In presence of a trigger sequence, complementary to the hairpin motif, the structure opens up, where distance between the cocatalysts is increased. As a result, synergistic catalysis is turned ‘*off*’. When a corresponding antitrigger sequence is added, the original catalyst conformation can be restored via formation of dsDNA as by product, and catalytic activity could be restored. The DNA-scaffolded cocatalysts exhibited activity in the oxidation of 6-methoxy-2-naphthalenemethanol (a fluorogenic probe). Thus, synergistic catalytic activity can be controlled through conformational switching of DNA triggered by chemical stimuli. Regulation of enzyme activity in response to environmental conditions is critical to many metabolic functions. Mimicking enzyme regulatory of nature, scientists are creating artificial nanoreactors for potential applications ranging from diagnostics to the production of artificial (smart) materials^144–146^. On this regard, chemistries involving protein–DNA conjugation provide the precession in positing proteins and other biomolecules on DNA scaffolds to modulate intermolecular interactions and the local environment^147–149^.

In nature, living systems have complex dissipative cellular networks, where advanced functions such as adaptability, responsiveness are feasible through energy consumption. Likewise, artificial dissipative chemical systems, which is out-of-equilibrium assemblies require inputs of energy in the form of chemical fuels^150–154^ or light^155,156^ to stay in a functional state. Out-of-equilibrium state of a synthetic molecular machine can be used to control catalysis^157^. In an interesting work, Maiti et al.^158^ demonstrated transient availability of vesicular nanoreactor for enhancing the rate of a nucleophilic aromatic substitution reaction. Assembly of the vesicle nanorecator was driven by the electrostatic complexation of anionic adenosine triphosphate (ATP) to a cationic surfactant, whereas potato apyrase enzyme hydrolyses ATP to adenosine monophosphate (AMP) leading to breakdown of the formed vesicles. The transient presence of the vesicles was used as nanoreactor to accelerate the reaction rate between 4-chloro-7-nitrobenzofurazan and 1-octanethiol in HEPES buffer (pH 7) as it provides suitable reaction environment, however, there is no catalyst is involved. In contrast, there is no effect on the reaction in presence of only the cationic surfactant or just ATP. This system could in principle be applied for many chemical reactions, because the only requirement is the hydrophobicity of the reactants^159^. Small biomolecule, such as ATP-induced temporal control over chemical reactivity in artificial synthetic system provided the dissipative catalyst systems as the basis for many events in biology^157,160–163^. In another example, Biagini et al.^164^ reported a molecular machine where rotaxane can be transiently changed from a catalytically inactive form into an active state by CCl_3_CO_2_H. Deprotonation by the rotaxane helped decarboxylation of the acid group, thereby bringing the system to its original state and stopping catalysis. However, the system works in organic solvent (CH_2_Cl_2_ or toluene). Recently, van der Helm et al.^165^ reported cucurbit[7]uril (CB[7]) as a supramolecular host to encapsulate aniline (catalyst molecule) in an aqueous environment, which can be released via addition of hydrolytically unstable esters as chemical fuel (Fig. 8d). Addition of glycine betaine methyl ester, as chemical fuel, resulted favorable binding toward CB[7], leading to expulsion of aniline from the cavity of CB[7]. In phosphate buffer (pH 7.5), aniline catalyzed hydrazone formation reaction between 2-formylbenzenesulfonate and a hydrazide derivative to form hydrazone product (Table 1, Entry 38). However, since the ester molecules are unstable under aqueous condition, they hydrolyze to corresponding acid and methanol, which are nonbonding to the cavity of CB[7]. As a result, aniline rebinds in the host cavity of CB[7], and switching ‘*off*’ the catalysis. Availability of aniline for catalyzing the reaction depends on the lifetime of ester molecule in aqueous environment. Thus, transient presence of a chemical fuel that can bind to a supramolecular host and expel a catalyst, can turn ‘*on*’ catalysis. An advantage of such catalytic system is that it could, in principle, be applied for many chemical reactions by altering the structure of catalyst and chemical fuel of different concentrations.

## Electrochemical-switchable catalytic reactions

**Fig. 9:**
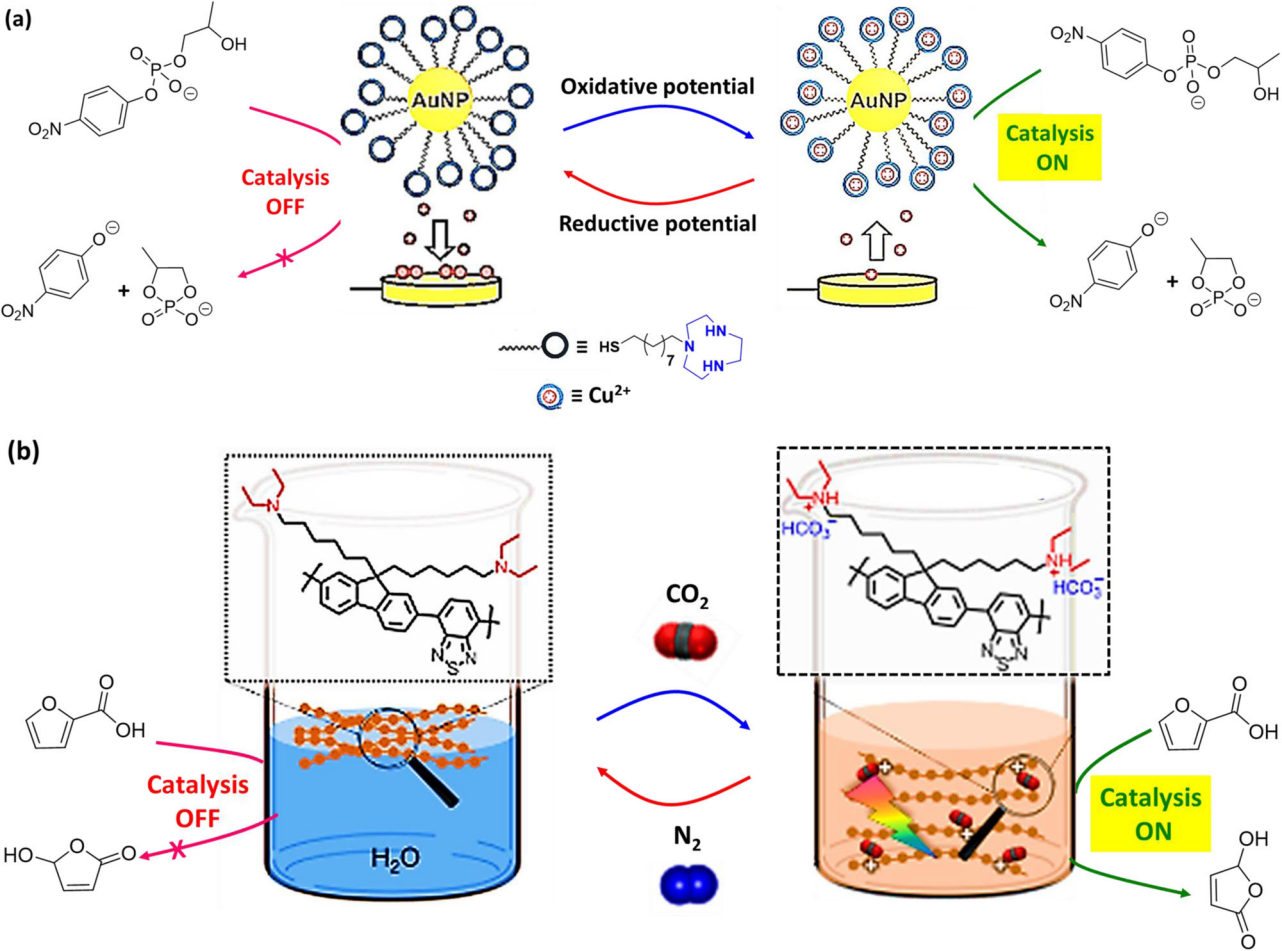
Other stimuli-switchable catalytic systems. **a** Electrochemical-induced switchable catalyst stem—activation and deactivation of Au-NP-based supramolecular nanocatalyst via electrochemical stimulus for the hydrolysis of HPNPP. Adopted with permission from the ref. ^170^, copyright 2016 WILEY-VCH Verlag GmbH & Co. KGaA, Weinheim. **b** Polymer photocatalyst via switchable hydrophilicity by CO_2_/N_2_ swing—in presence of CO_2_, photocatalyst generates a hydrophilic structure that enables the catalyst to support photo-oxidation of 2-furoic acid in water. On purging in N_2_ into the system, CO_2_ is released from the system and the photocatalysts clusters back to its original hydrophobic nature, terminating the catalytic activity and the reaction (inset: the structure of photocatalyst in absence/presence of CO_2_). Adopted with permission from ref. ^173^, copyright 2018 Wiley-VCH Verlag GmbH & Co. KGaA, Weinheim.

Application of electrochemical methods for synthesis of organic compounds in aqueous medium are very appealing from both an economic and an ecological point of view^166,167^. Electrochemical-induced activation/deactivation of inorganic and organic hybrid nanomaterials is a promising strategy as enzyme mimic for chemical transformations of organic molecules^168^. However, it is challenging to regulate these systems reversibly in aqueous environments. Au-NPs have been used for catalysis of transphosphorylation of HPNPP, which is a model compound used for mimicking RNA hydrolysis^169^. Employing electrochemical input, della Sala et al.^170^ reported reversible activation and deactivation of Au-NP-based supramolecular nanocatalyst in aqueous buffer (HEPES buffer, pH = 7.0). In this example, Au-NPs were covered with C_9_-thiols terminating with TACN head group and electrochemical controlled association/dissociation of metal ions have been used as a regulatory mechanism for catalysis (Fig. 9a). The electrochemical input in the form of oxidation potential generated Cu(II) coordinated Au-NP, which can switch on the transphosphorylation of HPNPP (Table 1, Entry 34). In contrast, application of reductive potential resulted Cu^2+^ → Cu^0^ conversion and redeposition onto the electrode surface. This provided copper-free Au-NP (inactive catalyst) to switch ‘*off*’ transphosphorylation of HPNPP. Likewise, electrochemistry can be used as effective and clean tool for the regulation of the catalytic activity of a supramolecular catalyst.

## Other stimuli-induced switchable catalytic systems

Multi stimuli-switchable systems in aqueous environments provide smart tunability in catalytic activity for organic transformations. In this regard, conjugated polymers, metal-organic frameworks have been employed by attaching stimuli-responsive functional groups for photocatalytic reactions in aqueous media^171,172^. In an example, Zhang et al.^173^ employed conjugated poly(bisthiophene) (PBT) for photocatalytic oxidation of 2-furoic acid into 5-hydroxy-2(5H)-furanone in aqueous medium (Table 1, Entry 39). In this example, PBT was functionalized with diethylamine (DEA), which is hydrophobic in nature. On introducing CO_2_ in the system, carbonic acid was generated and solubilized in aqueous medium, which can successfully perform visible-light-induced catalytic reactions (Fig. 9b). On purging the system with nitrogen, CO_2_ desorbs from the system and turns the polymeric segment back to its hydrophobic nature that stops the reaction and can be utilized to separate the product. Therefore, product formation is only feasible in presence of light and CO_2_ stimulus. The reversible switchability of polar nature for the polymeric segment in water was used for photocatalytic reduction of 4-nitrophenol to 4-aminophenol in presence of NaBH_4_ (Table 1, Entry 19), and visible-light-induced coupling of caffeine and aryldiazonium tetrafluoroborate as radical-mediated arylation of heteroarene (Table 1, Entry 40).

Application of magnetic field for controlled organocatalysis in water is scare. However, magnetic materials (nanoparticles) functionalized with organocatalysts have been employed for various organic transformations in aqueous environments due to their easy recovery and reuse^174,175^. In such systems, the magnetic core provides a magnetic function and the functionalized shell participates in catalysis. An interesting example exploiting thermoresponsive behavior of PNIPAM was described by Wang et al.^176^, where proline-based nanohybrid was prepared by grafting thermoresponsive PNIPAM fragment and magneto-sensitive vinyl modified Fe_3_O_4_@SiO_2_. In this example, PNIPAM fragment remained in dissolved state below the LCST (38 °C) due to the hydrophilic nature of PNIPAM and therefore catalytically inactive. In contrast, the PNIPAM turned hydrophobic at a higher temperature, resulting the nanohybrid aggregation to ‘*broom-type*’ corona that provided the hydrophobic microenvironment for catalyzing the aldol reaction of cyclohexanone and pNB (94% yield, 95% ee) (Table 1, Entry 15). Thereafter, magnetic responsiveness allowed to recover the nanohybrid material from the reaction mixture by application of an external magnetic field. Likewise, magnetic- and CO_2_-dual-responsive Pickering emulsion (PE)-based catalyst system was reported by Tang et al.^177^ employing Fe_3_O_4_ and DEA, which provides a switchable catalytic platform for the benzyl alcohol oxidation. Catalytic system was prepared by introducing DEA for CO_2_ responsiveness, Fe_3_O_4_ for magnetic responsiveness, and tetramethyl-4-piperidylmethacrylat (TMPM) as TEMPO precursor. As a biphasic reaction, the Anelli system (NaClO/NaBr/TEMPO)^178^ was employed for benzyl alcohol oxidation in water-in-oil PE. This catalyst system provided full conversion for benzyl alcohol (Table 1, Entry 37) and excellent yields (>94%) for other aromatic alcohols (diphenylmethanol, 2-phenylethanol, 4-nitrobenzyl alcohol) and aliphatic alcohols (cyclohexanol, 1-nonanol, 1-hexanol). Thereafter, bubbling of CO_2_ resulted protonation of the tertiary amino groups. As a consequence, demulsification happened due to formation of relatively hydrophilic tertiary amino groups, which desorbed from the interface and switching ‘*off*’ the catalysis. They can be quickly gathered under magnetic field, followed by purging of N_2_ results deprotonation of the tertiary amine group and formation of PE for reuse. Likewise, use of an external magnetic field provides the magnetic separation as a new way for separation of organocatalysts. However, in many examples, magnetic NPs (such as Fe_3_O_4_ and γ-Fe_2_O_3_) can catalyze a number of organic reactions due to its Lewis acid nature, besides the function as a magnetic core^179,180^.

Mechanical force could be employed as a stimulus for catalysis^181^. Generally, mechanical forces are applied in solution through the use of ultrasound^182^, and in solid state via collisions using milling balls^183^. However, reversible control over catalytic activity in aqueous environments using this stimulus is scare. Deactivation of catalytic species by generated radical during sonication and the requirement of large macromolecular chain that can be affected during sonication are the major limitations of using this stimulus for control catalytic activity. In addition, water can be only employed in liquid assisted grinding technique of milling ball approach^184^, but the process is not reversible. Nonetheless, more detailed insights for the mechanisms and processes underlying mechanochemical catalyst (de)activation can contribute to the rational design and implementation of switchable mechanocatalyzed systems in aqueous environments, with their potential use in smart application^185^.

## Conclusion and outlook

In conclusion, this account summarizes the advances in switchable catalytic systems in aqueous medium. Various “nature-like” catalyst systems including supramolecular aggregations, nanoreactors, and molecular catalysts have been enlisted. Taking inspiration from enzymatic activity in aqueous environments, reversible control over artificial catalytic systems for organic transformations has been challenging. However, significant advances have been made in the field of switchable aqueous catalytic systems either via improved catalyst design or via creation of a favorable microenvironment for the catalysis. The later approach is very similar in analogy to the natural enzyme, where hydrophobic pockets provide the optimal chemical environment. Artificial switchable catalysts can alter the reaction rate, selectivity in aqueous environments, but only a handful of organic transformations are successfully tested as benchmark reactions. Limited solubility of the substances and stability of the reaction intermediates are the typical drawbacks for “in water” organic transformations, whereas “on water” reactions can be performed for hydrophobic substances^15^. However, more strategies for creation of favorable active-site microenvironment via catalysis engineering should be developed, where both hydrophilic and hydrophobic substances can undergo synthetic transformations in aqueous environment, and the catalysis can be switched “*on*” and ‘*off*’ in presence/absence of stimuli. Besides, most efforts are concentrated on the invention of new types of switchable catalyst systems incorporating stimuli-responsive unit within their architecture for controlling over the catalytic activity. Moreover, majority of the catalytic systems have modest differences between the catalytic activities of ‘*on*’ and ‘*off*’ states of the systems. Although the field is in its infancy, artificial aqueous catalyst system requires development in the catalytic systems that accelerate the rate of reaction in the ‘*on*’ state and no reaction in the ‘*off*’ state. Besides, new chemical transformations with wider substrate scope should be tested.

Engineering over aqueous organocatalysis can be expected to grow in the coming years with a further broadening of its applications in biological environments. Besides stimuli-induced control over reaction rate, other characteristics such as substrate/product-selectivity have to be studied in switchable organocatalysis. It is worth noting that each stimulus comes with its own advantages and disadvantages for catalytic systems. As stimulus, temperature is employed mostly for switchable catalytic systems. These systems comprise PNIPAm or other polymers with different transition temperatures, whose efficiency can be switched in a wide range of temperatures. However, degradation at high temperature and probability of having side products are the limitation for temperature stimulus. Light has been used as noninvasive external trigger, which allows control in spatiotemporal precision. However, most of the examples are based on azobenzene-based molecules, while a larger variability could be obtained by employing different photoswitchable compounds. Electrochemically induced redox-switchable systems are convenient for metal-based catalysts due to easy modulation of redox-potential of the metal center. However, limitations regarding electrodes, and electrolytes remain major challenges in this field. With ongoing technological advancement, the scope, utility, and versatility of various stimuli-induced aqueous catalysis for organic transformations will continue to grow^186^. Altogether, a bright future is ahead for smart organocatalytic processes and encourage their applications in biological systems.
